# Flufenamic acid improves survival and neurologic outcome after successful cardiopulmonary resuscitation in mice

**DOI:** 10.1186/s12974-022-02571-2

**Published:** 2022-09-01

**Authors:** Jiancong Chen, Yuan Chang, Juan Zhu, Yuqin Peng, Zheqi Li, Kunxue Zhang, Yuzhen Zhang, Chuman Lin, Zhenzhou Lin, Suyue Pan, Kaibin Huang

**Affiliations:** Department of Neurology, Nanfang Hospital, Southern Medical University, Guangzhou North Avenue, Guangzhou, 1838#510515 China

**Keywords:** Blood–brain barrier, Cardiac arrest, Flufenamic acid, Microglia/macrophage, Transient receptor potential M4

## Abstract

**Background:**

Brain injury is the main cause of high mortality and disability after successful cardiopulmonary resuscitation (CPR) from sudden cardiac arrest (CA). The transient receptor potential M4 (TRPM4) channel is a novel target for ameliorating blood–brain barrier (BBB) disruption and neuroinflammation. Herein, we tested whether flufenamic acid (FFA), which is reported to block TRPM4 with high potency, could confer neuroprotection against brain injury secondary to CA/CPR and whether its action was exerted by blocking the TRPM4 channel.

**Methods:**

Wild-type (WT) and *Trpm4* knockout (*Trpm4*^*−/−*^) mice subjected to 10-min CA/CPR were randomized to receive FFA or vehicle once daily. Post-CA/CPR brain injuries including neurologic deficits, survival rate, histological damage, edema formation, BBB destabilization and neuroinflammation were assessed.

**Results:**

In WT mice subjected to CA/CPR, FFA was effective in improving survival and neurologic outcome, reducing neuropathological injuries, attenuating brain edema, lessening the leakage of IgG and Evans blue dye, restoring tight junction protein expression and promoting microglia/macrophages from the pro-inflammatory subtype toward the anti-inflammatory subtype. In comparison to WT mice, *Trpm4*^*−/−*^ mice exhibited less neurologic deficiency, milder histological impairment, more BBB integrity and more anti-inflammatory microglia/macrophage polarization. As expected, FFA did not provide a benefit of superposition compared with vehicle in the *Trpm4*^*−/−*^ mice after CA/CPR.

**Conclusions:**

FFA mitigates BBB breach and modifies the functional status of microglia/macrophages, thereby improving survival and neurologic deficits following CA/CPR. The neuroprotective effects occur at least partially by interfering with the TRPM4 channel in the neurovascular unit. These results indicate the significant clinical potential of FFA to improve the prognosis for CA victims who are successfully resuscitated.

**Supplementary Information:**

The online version contains supplementary material available at 10.1186/s12974-022-02571-2.

## Introduction

Sudden cardiac arrest (CA) is a leading cause of global mortality [1]. Despite many advances in optimizing the techniques of cardiopulmonary resuscitation (CPR), the overall prognosis is still unsatisfactory after out-of-hospital CA with successful resuscitation, mainly due to post-CA syndrome [2, 3]. Brain injury represents an essential hallmark of the pathophysiology of post-CA syndrome [4], profoundly impairing neurological function and leading to lifelong disability and even death among CA survivors. Nevertheless, no clinically effective pharmacological intervention is available to reduce neurologic deficiency in patients with CA/CPR at present [5].

Currently, most neuroprotective methods after CA/CPR have focused on protecting neurons. However, endothelial dysfunction, an inevitable pathologic process of ischemia/reperfusion during CA plays a governing role in the progression of post-CA brain injury and evokes further neuronal damage [6]. Blood–brain barrier (BBB) integrity is compromised by CA/CPR, leading to intractable cerebral edema. More significantly, cerebral edema exacerbates clinical outcomes and is a strong prognostic factor of outcomes after CA [7–9]. Microglia/macrophages are spectacularly plastic and obtain multiple subtypes, such as pro-inflammatory and anti-inflammatory subtypes, to fulfill different activities in health and disease. Altered microglia/macrophages functional subtypes and consequent neuroinflammation have an intimate relationship with the integrity of the BBB. The unwanted entry of serum proteins into the brain after the collapse of the BBB is a pivotal cause of neuroinflammation [10], facilitating the transition of microglia/macrophages to a pro-inflammatory status. The pro-inflammatory phenotype of microglia, in turn, can simultaneously exaggerate BBB insult [11]. In this way, a vicious cycle between BBB breakdown and pro-inflammatory microglia/macrophage status is created to continuously escalate post-CA/CPR brain injury. Therefore, only drugs that modulate multiple targets, such as BBB destruction, neuroinflammation and neuronal injury, are likely to achieve clinical translation. However, the molecular mechanisms responsible for controlling the restrictive feature of the BBB and microglia/macrophage polarization in post-CA/CPR brain injury remain largely elusive.

Transient receptor potential M4 (TRPM4), a nonselective monovalent cation channel activated by elevated intracellular Ca^2+^, has been shown to be critical in regulating BBB function [12]. The findings of studies conducted by us and others indicated that pharmacological inhibition of the subunits of sulfonylurea receptor 1-transient receptor potential M4 (SUR1-TRPM4) or gene deletion of *Trpm4* could function in preserving BBB integrity in animal models of status epilepticus [13], acute ischemic stroke [14], spinal cord injury [15], intracerebral hemorrhage [16], and subarachnoid hemorrhage [17]. Indeed, we have reported that glibenclamide, a selective inhibitor of SUR1, effectively improved survival and neurologic outcome in rodent models of CA/CPR [18–20], but its protective effect on the BBB after CA/CPR and whether it works by blocking the SUR1-TRPM4 channel remain unclear. Furthermore, the SUR1-TRPM4 channel was shown to be expressed in microglia and participated in regulating pro-inflammatory gene expression [21]. However, whether the TRPM4 channel can affect microglia polarization has not yet been understood. We hypothesized that interfering with the TRPM4 channel may therapeutically manipulate BBB function and the phenotypic shift of microglia/macrophages, which could serve as a promising opportunity for minimizing brain injury resulting from CA/CPR.

Flufenamic acid (FFA) is a non-steroidal anti-inflammatory drug that has been applied for analgesia for pain related to rheumatic disorders. Since the TRPM4 channel was found to be highly sensitive to FFA and can be inhibited by low concentrations of FFA, FFA has attracted extensive attention as a convenient and relatively selective TRPM4 inhibitor to study the physiological effects of TRPM4 [22, 23]. Emerging studies have suggested that FFA confers neuroprotection against several neurological diseases, such as spinal cord injury [24], Alzheimer’s disease [25], and epilepsy [26]. FFA inhibited capillary fragmentation and secondary hemorrhage by blocking TRPM4 after spinal cord injury [24]. However, the protective effects of FFA on brain injury secondary to CA/CPR and the underlying mechanisms have not been addressed. These studies prompted us to logically postulate that FFA could elicit its neuroprotective effects after CA/CPR by ameliorating BBB breakdown and improving neurologic outcome by inhibiting TRPM4.

Here, we investigated whether FFA could improve neurologic outcome in a mouse model of CA/CPR. Moreover, we aimed to explore whether gene deletion of *Trpm4* (*Trpm4*^*−/−*^) could exert an effect similar to that of FFA and whether TRPM4 blockage represents part of the mechanism accounting for FFA-mediated neuroprotection in CA/CPR.

## Materials and methods

### Animals

All experiments were approved by the Animal Care and Use Committee of Nanfang Hospital, Southern Medical University and adhered to the National Institutes of Health Guide for the Care and Use of Laboratory Animals. Animal data are reported in accordance with ARRIVE guidelines. In the first part of the study, male wild-type (WT) C57BL/6J mice (8–12 weeks, 25–30 g) were purchased from the Experimental Animal Center of Southern Medical University. In the second part, male *Trpm4*^*−/−*^ mice on a C57BL/6J background (8–12 weeks, 25–30 g, Shanghai Model Organisms China) and WT littermates were bred from homozygous mating. All mice were housed in the specific pathogen-free facility with a 12-h/12-h light/dark cycle and provided free access to water and standard chow. The genotypes of the mice were confirmed by extracting DNA from mouse tails and then separating the DNA in agarose gels (Additional file 1: Fig. S1). All efforts were made to minimize the number of animals used and their suffering in this study.

### Mouse model of CA/CPR

**Fig. 1:**
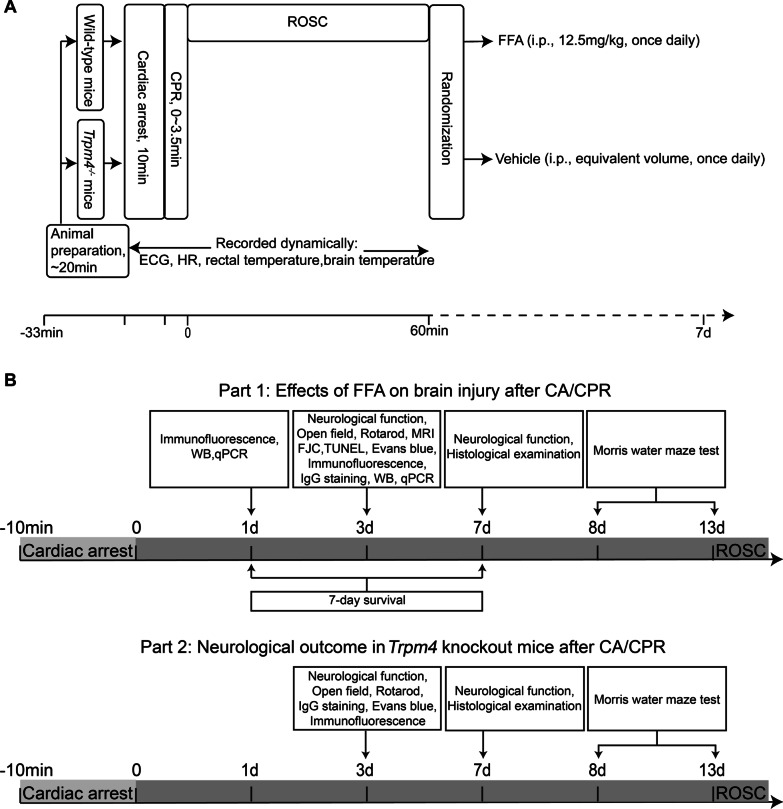
Experimental procedures and flow diagram of the study. **A** Experimental procedures, drug delivery, and measurements during baseline, cardiac arrest and cardiopulmonary resuscitation and ROSC. **B** Flow diagram for the study assessments on a timeline. The whole study consisted of 2 parts. HR: heart rate; i.p.: intraperitoneal injection; WB: Western blot

The CA/CPR model in mice was established as previously described with minor modifications (Fig. 1A) [27]. In brief, mice were anesthetized, endotracheally intubated with a 22 G cannula and connected to a mouse ventilator. Normothermia was maintained during the surgery. Internal jugular venous catheters were placed for drug delivery. Electrocardiograms (ECGs) were monitored with subcutaneous needle electrodes throughout the surgical procedure. After 5-min stabilization, CA was induced by intravenous administration of potassium chloride (50 µL, 0.5 M) and was defined as the appearance of isoelectric tracing on the ECG monitor. During CA, the ventilator was disconnected, and the pericranial temperature was maintained at 38.5 ± 0.2 °C. CPR was initiated at the end of 10-min CA by mechanical ventilation with 100% oxygen, the injection of epinephrine (0.5 mL, 16 µg/mL) and finger chest compressions at a rate of 300 compressions per minute. The return of spontaneous circulation (ROSC) was defined as stable spontaneous electrical activity on ECG. Mice failed to ROSC within 3.5 min were excluded from the subsequent experiments. Once spontaneous respiration was confirmed, mice were extubated and surgical wounds were sutured. At the end of each experimental period, mice were placed in their cages with easy access to food and water.

### Experimental design

The present experiment was divided into two parts (Fig. 1B). In part 1, the effects of FFA on CA/CPR-treated WT mice were investigated. At 1 h after ROSC, mice were randomized to receive FFA or vehicle by using a random number table (Fig. 1A). Mice in the FFA group were given FFA (12.5 mg/kg in sterile saline, 4% PEG-400, 2% DMSO; Sigma, St. Louis, MO) via intraperitoneal injection once daily for 1 week [24], while mice in the vehicle group received an equivalent volume of vehicle solution. Mice that were successfully resuscitated were followed up for 7 days to evaluate survival, behavioral tests, and histological injuries. The other mice were used to determine the neuroprotective effects by evaluating cellular apoptosis, neuronal degeneration, BBB disruption, edema formation and neuroinflammation markers at day 3 after CA/CPR. In part 2, we explored whether gene deletion of *Trpm4* improved neurologic outcome after CA/CPR and whether FFA interfered with the TRPM4 channel. *Trpm4*^*−/−*^ mice that underwent CA/CPR were randomly allocated to receive vehicle or FFA, and CA/CPR-treated WT mice given vehicle were set as the control group (Fig. 1A). In this part, the expression of TRPM4 was evaluated at 24 h after CA and other experiments were in line with those of part 1.

### Survival study and neurologic outcome assessment

Survival was recorded daily for 7 days after CA/CPR. A previously reported neurological function scoring system was utilized by two investigators (who were blinded to the animal grouping) to assess neurologic deficiency at day 3 and day 7 after ROSC in all successfully resuscitated animals [28]. The scoring system consisted of five parameters: consciousness, corneal reflex, respiration, coordination, and movement/activity. A total score of 10 was considered normal.

### Behavioral analysis

#### Rotarod

Motor coordination of mice was evaluated at day 3 after CA/CPR by the rotarod test. Mice were trained for 3 days before CA/CPR. During the test, the mice were gently placed on the rotarod cylinder. The rotating speed was gradually accelerated to 40 rpm over the course of 5 min. There were two trials per subject including a 30 min break in between. The time remaining on the rotating rod was recorded, and the mean duration was used for comparison among groups.

#### Open field test

We used the open field test to examine gross motor function and general activity in mice at day 3 after CA/CPR. Briefly, mice were placed into a 50 × 50 cm field surrounded by 50 cm high walls to enable them to explore the box freely for 10 min. The total distance covered by mice was recorded using an animal behavior analysis system (VIDEOMOT 2, TSE, Germany).

#### Morris water maze test

The Morris water maze test was designed at day 8 after CA/CPR to assess the spatial learning and memory capability of mice, including spatial exploration experiments and directional navigation experiments. The Morris water maze test was performed as described previously [13]. In the spatial exploration experiments, mice were placed in the water from four starting points in four quadrants, and the time required to locate the hidden platform within 60 s was recorded as the escape latency. If the mice did not find the platform in 60 s, the escape latency was recorded as 60 s, and the mice were guided to the platform and allowed to stay there for 15 s. These steps were repeated for five consecutive days to accomplish the hidden platform training. In the directional navigation experiments, the platform was removed, and the mice were allowed to search the pool freely for 60 s. The number of platform location crossings and the percentage of time spent in the target quadrant were measured. During the test, all data were recorded using an animal behavior analysis system (VIDEOMOT 2, TSE, Germany).

#### Immunofluorescence

Deeply anesthetized mice were perfused with ice-cold saline and 4% paraformaldehyde and the brain was dissected. After post-fixation with 4% paraformaldehyde for 24 h, the brains were immersed in 15% sucrose and 30% sucrose. Cryosections (6 μm or 20 μm) were obtained by slicing with Cryostat Microtome (Leica CM1800, Leica Microsystems GmbH, Germany). For immunofluorescence, slices were permeabilized with 0.5% Triton X-100 and blocked with 2% bovine serum albumin, followed by overnight incubation at 4 °C with primary antibodies involving: anti-neuronal nuclei (NeuN; Proteintech, Chicago, IL), anti-ionized calcium-binding adapter molecule 1 (Iba1; Abcam, Cambridge, UK), anti-glial fibrillary acidic protein (GFAP; Proteintech), anti-microtubule-associated protein 2 (MAP2; Abcam), anti-CD31 (Abcam), anti-ZO-1 (Invitrogen, Carlsbad, CA), anti-occludin (Invitrogen), anti-claudin-5 (Invitrogen), anti-intercellular cell adhesion molecule-1(ICAM-1, Santa Cruz, CA, USA), anti-Arg1 (Santa Cruz), anti-CD16/32 (Invitrogen) and anti-TRPM4 (Sigma-Aldrich). Then, slices were washed and incubated with the appropriate Alexa Fluor dye of secondary antibodies for 1 h at 37 °C. DAPI was used to stain the nucleus. Fluorescence images were randomly obtained with a fluorescence microscope (LSM 880, Carl Zeiss, Germany). The relative intensity area of MAP2, GFAP, occludin, claudin-5, ZO-1, CD31, and ICAM-1 staining and the number of cells with immunoreactivity for the other above markers were analyzed blindly by independent investigators using ImageJ software.

#### Fluoro-Jade C staining

For the evaluation of neuronal degeneration, Fluoro-Jade C (FJC) staining was performed with an FJC ready-to-dilute staining kit (Biosensis, USA). Slides were incubated with 1% sodium hydroxide in 80% ethanol for 5 min. After washing with 70% ethanol for 2 min and then with distilled water for 2 min, the slides were incubated with 0.06% potassium permanganate solution for 10 min. Slides were rinsed in distilled water for 2 min, followed by being transferred into a 0.0001% solution of FJC dissolved in 0.1% acetic acid. Afterward, slides were rinsed in distilled water for 1 min in each of 3 distilled water rinses and dried at 60 °C for 10 min. Finally, slides were cleared in xylene for 1 min and cover-slipped with DPX. The number of FJC-positive cells was quantified by independent investigators using ImageJ software.

#### Determination of cellular apoptosis

For the detection of cellular apoptosis, slices were stained with terminal deoxynucleotide transferase-mediated dUTP-biotin nick-end labeling (TUNEL) using an in situ cellular apoptosis detection kit (Meilunbio, Dalian, China) [18]. Briefly, slices were incubated with TUNEL at 37 °C for 1 h, washed with PBS three times and then mounted and observed with a fluorescence microscope. The number of TUNEL-positive cells was analyzed blindly by independent investigators using ImageJ software.

#### Evaluation of BBB permeability by staining with IgG and Evans blue

To detect the integrity of the BBB, immunofluorescence staining was performed with an anti-mouse IgG secondary antibody [29]. The relative intensity of IgG leakage was blindly obtained with ImageJ software.

Evans blue (2% in saline, 4 ml/kg, Sigma-Aldrich, USA) dye was administered intravenously. After 1 h, the mice were perfused with saline to wash out intravascular dye. The brains of mice were removed and immersed in methanamide (Macklin, Shanghai, China) and soaked at 60 °C overnight. The concentration of Evans blue was quantified using a microplate reader (SpectraMax M5, Molecular Devices, Sunnyvale).

#### MRI

MRI was performed at 72 h after ROSC as described previously [30]. During MRI, mice were anesthetized, and anesthesia was maintained with inhaled isoflurane (5% induction, 1–2% maintenance). The brains were scanned from the olfactory bulb to the brainstem by a 7.0 Tesla horizontal-bore magnet (PharmaScan 70/16 US, Bruker). A diffusion-weighted imaging (DWI) method sequence was obtained. To quantify brain edema after CA/CPR, average ADC values in the lateral hippocampus, striatum and cortex were calculated by a researcher blinded to group allocation.

#### RNA preparation and RT-PCR

The total RNA of brain tissues was isolated by using TRIzol (Takara, Kusatsu, Japan) and then reverse-transcribed to cDNA using the PrimeScript™ RT Master Mix Kit (Takara). RT-PCR was conducted with SYBR Green master mixes (Takara) on a Roche LightCycler 480 System to measure the mRNA levels of *Trpm4*, *interleukin (IL)-1β*, *tumor necrosis factor-α (TNF-α)*, *inducible nitric oxide synthase (iNOS)*, *transforming growth factor-β (TGF-β)*, *Arg1*, *IL-10* and *glyceraldehyde phosphate dehydrogenase (Gapdh)*. Relative changes in mRNA expression were normalized to *Gapdh* [19].

#### Western blot

Western blotting was routinely performed as previously reported [31]. Briefly, brain tissues were quickly dissected on ice and placed in RIPA lysis buffer (Beyotime, Shanghai, China) with protease inhibitor cocktail and phosphatase inhibitor (Beyotime, Shanghai, China). Denatured proteins in SDS-loading buffer were separated on SDS-PAGE gels and transferred to polyvinylidene difluoride membranes (Millipore, Billerica, United States). After blocking, the membranes were incubated overnight at 4 °C with primary antibodies involving anti-occludin (Invitrogen), anti-ZO-1 (Invitrogen), anti-TNF-α (Santa Cruz), anti-iNOS (Proteintech), anti-Arg1 (Santa Cruz), anti-TRPM4 (Sigma-Aldrich) and anti-β-actin (Proteintech). After washing with TBST, membranes were incubated with HRP-conjugated secondary antibodies (Proteintech), and bands were detected by enhanced chemiluminescence advance Western blotting detection reagents (FDbio, Hangzhou, China). The intensities of the protein bands were quantified and normalized to the level of β-actin by using ImageJ software.

### Statistical analysis

Preliminary analysis of data normality was performed with Shapiro–Wilk’s test. All data are expressed as the mean ± SD or medians and 25th to 75th percentiles (neurological function scores). The comparison of data between two groups was performed with two-tailed *t*-test while the comparison of data among multiple groups was performed by one-way ANOVA followed by Tukey’s post hoc multiple comparison tests. Differences in survival rate were examined by the log-rank test. Neurological function scores were compared with the Mann–Whitney *U* test or Kruskal–Wallis test with Dunn's multiple comparisons test. Statistical analysis was conducted using GraphPad Prism Version 6.0 and SPSS 20.0. *P* < 0.05 was considered statistically significant.

## Results

### FFA improved survival and neurologic outcome after CA/CPR

There were no differences in body weight, the time required for ROSC, the total epinephrine dose, and heart rate among the experimental groups (Additional file 1: Tables S1, S2). The partial pressure of oxygen and oxygen saturation of arterial blood samples obtained at 2 h after CPR did not differ among the experimental groups (data not shown). There was no difference in core body temperature among the experimental groups for 1 h after CA/CPR (data not shown).

**Fig. 2:**
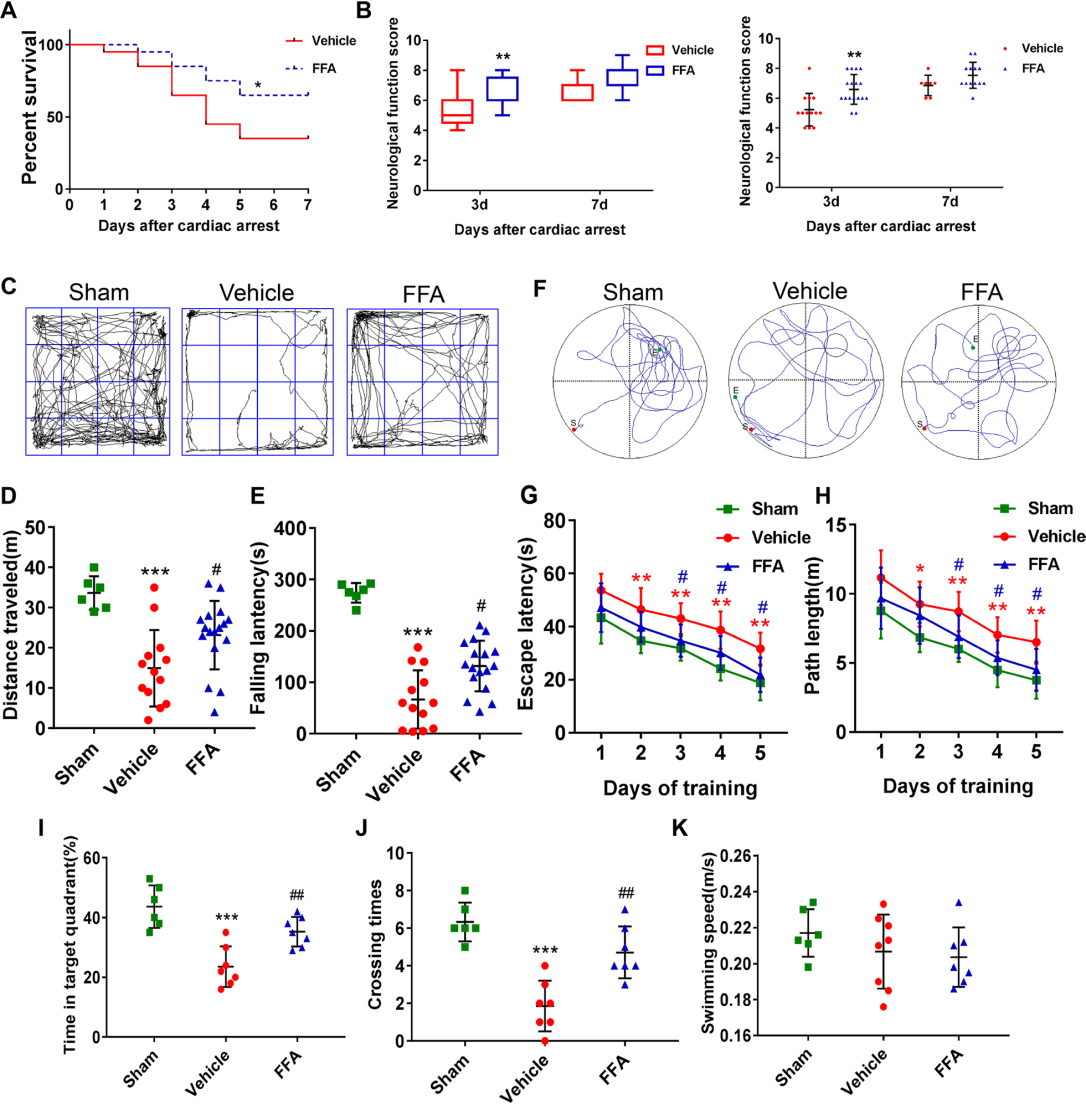
Effects of FFA on survival and neurological function after CA/CPR. **A** Kaplan–Meier analyses of cumulative survival during the 7-day follow-up after ROSC. Solid line, vehicle group (*n* = 20); Dashed line, FFA group (*n* = 20). **B** Neurological function scores of survived mice at 3 days and 7 days after ROSC. *P < 0.05, **P < 0.01 versus the vehicle group. **C** Representative paths of mice during the open field test. **D** The total distance traveled in the open field test. **E** The latency to drop from the accelerating rotarod. **F** Representative swimming paths of mice during the probe trial. The latency to find the platform (**G**) and swimming distance (**H**) during the spatial exploration experiment. The percentage of time spent in the target quadrant (**I**) and platform crossovers (**J**) during the probe trial. (**K**) The swimming speed of mice in the Morris water maze test. **P < 0.01, ***P < 0.001 versus the sham group (*n* = 6); #*P* < 0.05, ##*P* < 0.01 versus the vehicle group

We firstly observed the survival rate during 7 days after CA/CPR, and the results revealed that 35% of mice (7 of 20) survived in the vehicle group, compared with 65% of mice (13 of 20) in the FFA group, until day 7 at the end of the study (*P* = 0.0471) (Fig. 2A). Furthermore, we used a 10-point scoring system to assess the overall neurologic deficits and found that FFA-treated mice exhibited statistically higher neurological function scores at 3 days after ROSC (Fig. 2B). Nonetheless, no apparent difference was found in the neurologic score at 7 days between the vehicle and FFA groups, possibly because the animals with more severe neurological deficits in the vehicle group had died. Additionally, post-CA mice in the FFA group displayed significantly better performance in both the rotarod test and the open field test at 3 days after ROSC (Fig. 2C–E).

The effect of FFA on cognitive function after CA/CPR was further evaluated by the Morris water maze test. During the spatial exploration experiment on day 8 to day 12 after CA/CPR, mice in the vehicle group spent more time searching for the hidden platform and had a longer swimming path length than mice in the sham group, whereas FFA treatment improved spatial memory, presenting as significantly decreased latency and swimming distance to find the hidden platform (Fig. 2G, H). In the probe trial on day 13, the vehicle group showed shorter target quadrant traveling time and platform crossing time than the sham group. Moreover, FFA administration markedly increased the crossovers of the previous platform area and the time spent in the target quadrant in comparison with the vehicle group (Fig. 2F, I, J). Besides, the swimming speed was comparable among the three groups, which demonstrated that the different performances in the Morris water maze test were not attributed to motor impairment (Fig. 2K). On the basis of these data, we suggest that FFA treatment improved cognitive dysfunction after CA/CPR.

Collectively, these data indicated that post-CA treatment with FFA resulted in better 7-day survival and functional outcome than treatment with vehicle.

#### FFA alleviated neuronal loss and suppressed the activation of glial cells following CA/CPR

**Fig. 3:**
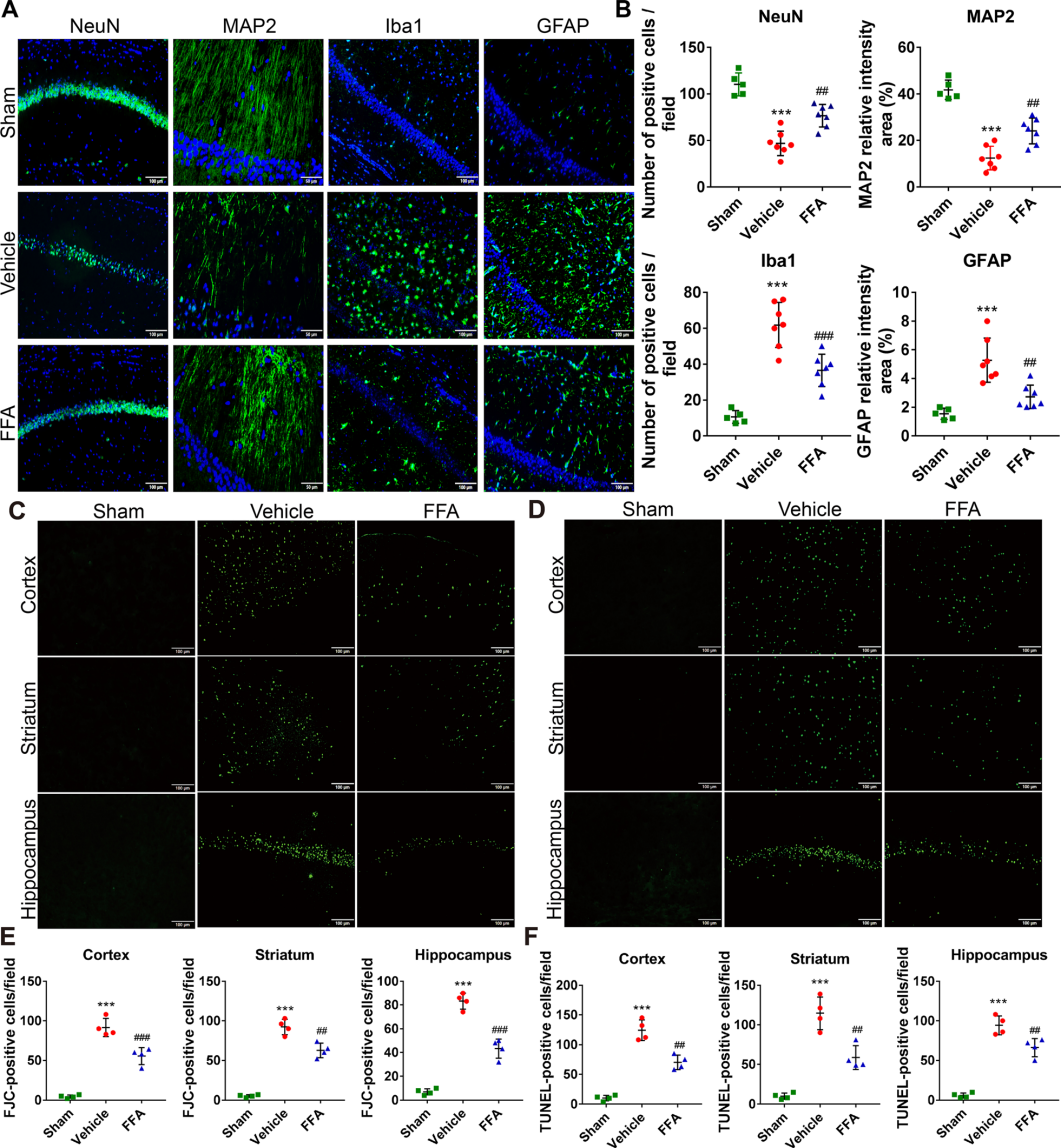
Effects of FFA on neuropathological damage after CA/CPR. **A** Representative photomicrographs of post-cardiac arrest neuropathological damage characterized by immunofluorescence staining for NeuN, MAP2, Iba1, and GFAP in the hippocampal CA1 region of the sham group and the experimental groups at 7 days after ROSC. Scale bar, 100 μm or 50 μm. **B** Semi-quantitative results of NeuN, MAP2, Iba1, and GFAP staining. Representative photomicrographs of Fluoro-Jade C (FJC) staining (**C**) and terminal deoxynucleotide transferase-mediated dUTP-biotin nick-end labeling (TUNEL) staining (**D**) in the cerebral cortex, striatum, and hippocampal CA1 region of the sham group and the experimental groups at 3 days after ROSC. Scale bar, 100 μm. **E**, **F** Quantification of FJC-positive and TUNEL-positive cells, respectively. ***P < 0.001 versus the sham group; ##*P* < 0.01, ###*P* < 0.001 versus the vehicle group, *n* = 4–7

After the transient global cerebral ischemia and reperfusion induced by CA/CPR, delayed neuronal injury occurs in selectively vulnerable regions, such as the hippocampal CA1 region [32]. Therefore, we conducted immunofluorescence staining to observe the differences in neuronal loss among the different groups. The results showed that the number of surviving neurons stained by NeuN was significantly reduced in the vehicle group compared with the sham group, and this neuronal loss was substantially mitigated by FFA treatment (Fig. 3A, B). Additionally, CA/CPR led to widespread injury to neuronal dendrites, as evidenced by obviously decreased MAP2 immunostaining compared to the sham group, and this dendritic injury was partly averted by FFA treatment (Fig. 3A, B).

Microglia and astrocytes play important roles in orchestrating the neuroinflammatory response. They are activated and migrate to the injury site and thereby cause delayed neuron loss following CA/CPR. As illustrated, significantly more Iba1-positive microglia and GFAP-positive astrocytes were detected in the vehicle group than the sham group (Fig. 3A, B), indicating that neuroinflammation was activated in the brain after CA/CPR. On the other hand, FFA treatment notably suppressed the activation of microglia and astrocytes compared with vehicle treatment (Fig. 3A, B). Together, these results showed that FFA treatment markedly attenuated histological impairment in post-CA mice.

#### FFA mitigated CA/CPR-induced neuronal degeneration and cellular apoptosis in the brain

To validate whether FFA treatment could ameliorate neuronal degeneration, we carried out FJC staining to confirm the degeneration of neurons at 3 days after CA/CPR. The results revealed that CA/CPR induced an extensive degenerative reaction in the cortex, striatum, and hippocampus, which was partly preserved by FFA treatment (Fig. 3C, E). We proceeded to evaluate cellular apoptosis in the brain at 3 days after CA/CPR in the different groups by TUNEL staining. Compared with the sham group, robust cellular apoptosis was detected in the cortex, striatum, and hippocampus, whereas FFA treatment decreased the number of TUNEL-positive cells (Fig. 3D, F). In summary, these findings suggested that FFA treatment suppressed neuronal degeneration and cellular apoptosis in the brains of mice subjected to CA/CPR.

#### FFA prevented brain edema following CA/CPR

**Fig. 4:**
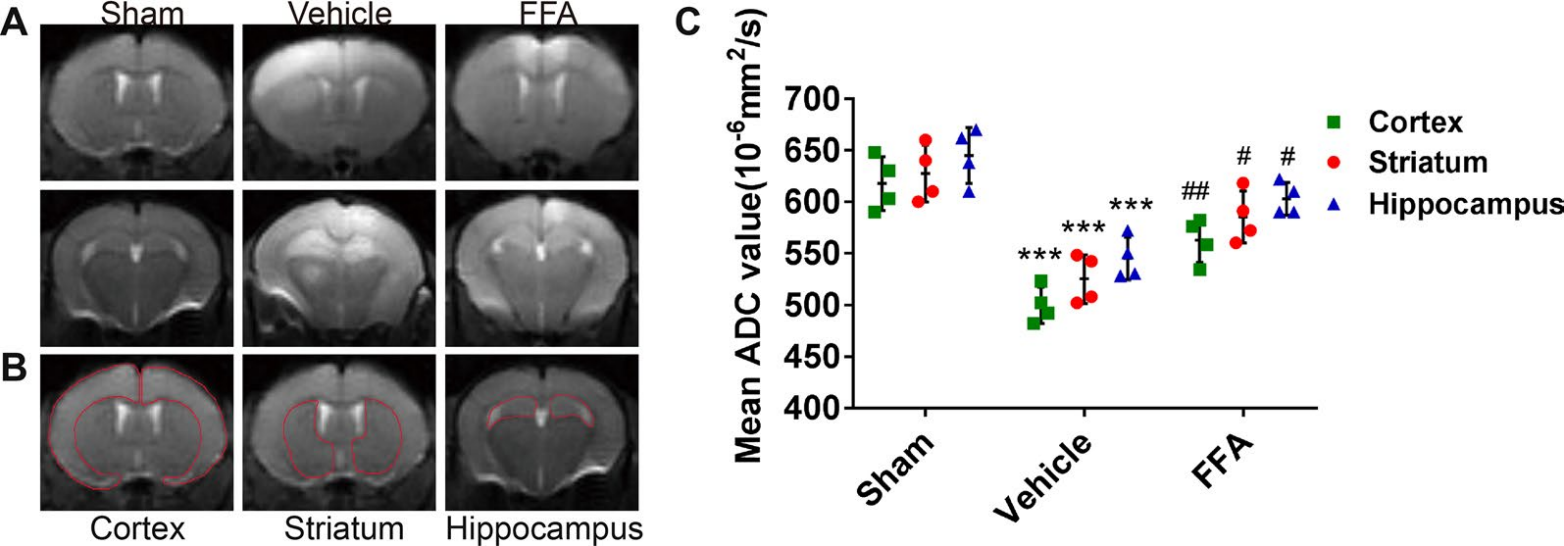
Effects of FFA on brain edema formation after CA/CPR. **A** DWI of the sham group and the experimental groups at 3 days after ROSC. **B** Representative DWI showing three slices containing regions of interest. **C** Quantification of average ADC values in the cortex, striatum, and hippocampus to detect the degree of abnormal water diffusion. ***P < 0.001 versus the sham group; #*P* < 0.05, ##*P* < 0.01 versus the vehicle group, *n* = 4

Considering that brain edema is a key modulator of outcome after brain injury from CA/CPR, we wondered whether FFA treatment would have any influence on CA-evoked brain edema. Cranial MRI was employed to visualize cerebral edema at 72 h following CA/CPR. As shown, brain edema was prominent in the vehicle-treated mice, whereas it was visibly prevented by FFA treatment (Fig. 4A). The degree of abnormal water diffusion was quantified by calculating the ADC values in regions of interest, including the cortex, striatum, and hippocampus (Fig. 4B). FFA alleviated the reduction in ADC values in each region of interest (Fig. 4C). The above data illustrated that the alleviation of cerebral edema could be attributed to FFA-afforded neuroprotection against CA/CPR.

#### FFA attenuated BBB disruption after CA/CPR

**Fig. 5:**
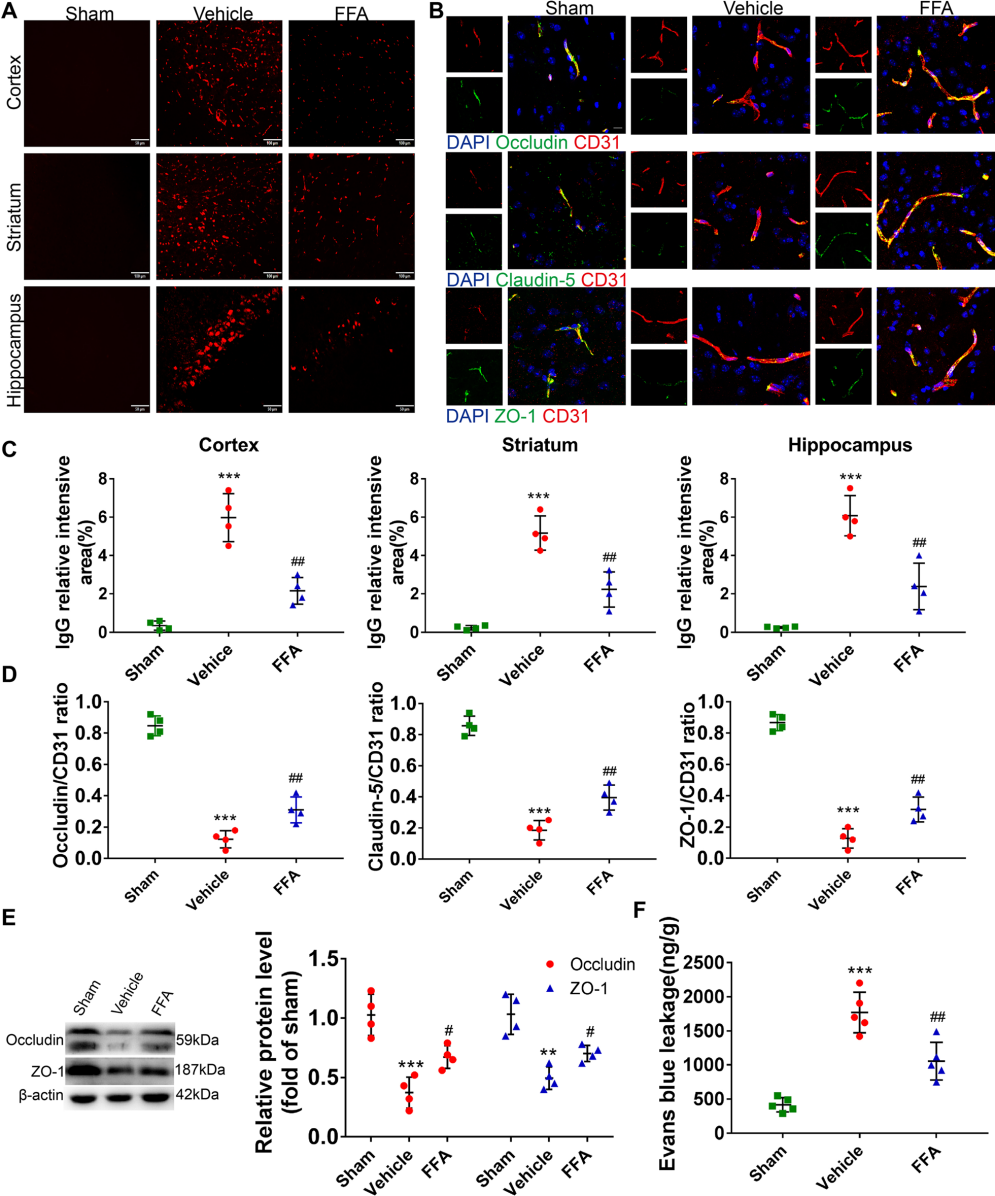
Effects of FFA on BBB integrity after CA/CPR. **A** Representative photomicrographs of immunofluorescence staining with IgG antibody (red) in the cerebral cortex, striatum and hippocampus of the sham group and the experimental groups at 3 days after ROSC. Scale bar, 100 μm or 50 μm. **B** Representative photomicrographs of immunofluorescence staining for tight junction proteins (green), including Occludin, Claudin-5, ZO-1, and CD31 (red) with DAPI (blue) in the brains of the sham group and the experimental groups at 3 days after ROSC. Scale bars, 10 μm. **C** Semi-quantitative results of IgG leakage. **D** Quantitative analysis of the Occludin/CD31 ratio, Claudin-5/CD31 ratio and ZO-1/CD31 ratio. **E** Western blot analysis of tight junction proteins in the sham group and the experimental groups at 3 days after ROSC. **F** Quantification of Evans blue leakage in the sham group and the experimental groups at 3 days after ROSC. **P < 0.01, ***P < 0.001 versus the sham group; #*P* < 0.05, ##*P* < 0.01 versus the vehicle group, *n* = 4–5

To further characterize the mechanism of neuroprotection by FFA treatment, the severity of BBB disruption, a crucial cause and marker of brain edema, was examined using an IgG extravasation assay in brain sections and Evans blue dye leakage. As illustrated, visible IgG penetration into the cortex, striatum, and hippocampus following CA/CPR was observed, but it was remarkably reduced after treatment with FFA (Fig. 5A, C). Besides, FFA decreased the leakage of Evans blue dye (Fig. 5F).

Since endothelial tight junctions are major components in maintaining the normal barrier function of the BBB, we next measured the expression levels of Occludin, Claudin-5 and ZO-1 by immunofluorescence staining. Compared with sham operation, the fluorescence intensity of Occludin, Claudin-5 and ZO-1 covering the brain vessels in the vehicle-treated post-CA mice was significantly reduced, and FFA treatment partially reversed the decrease in fluorescence intensity (Fig. 5B, D). Consistently, Western blot analysis demonstrated that the CA/CPR-induced reduction in Occludin and ZO-1 was largely reversed by FFA (Fig. 5E). Overall, our results clearly demonstrated that FFA could restore BBB function, which was partly due to the protection of the endothelial tight junctions following CA/CPR.

#### FFA downregulated pro-inflammatory cytokine expression and upregulated inflammation resolution-associated molecule expression in the brain

**Fig. 6:**
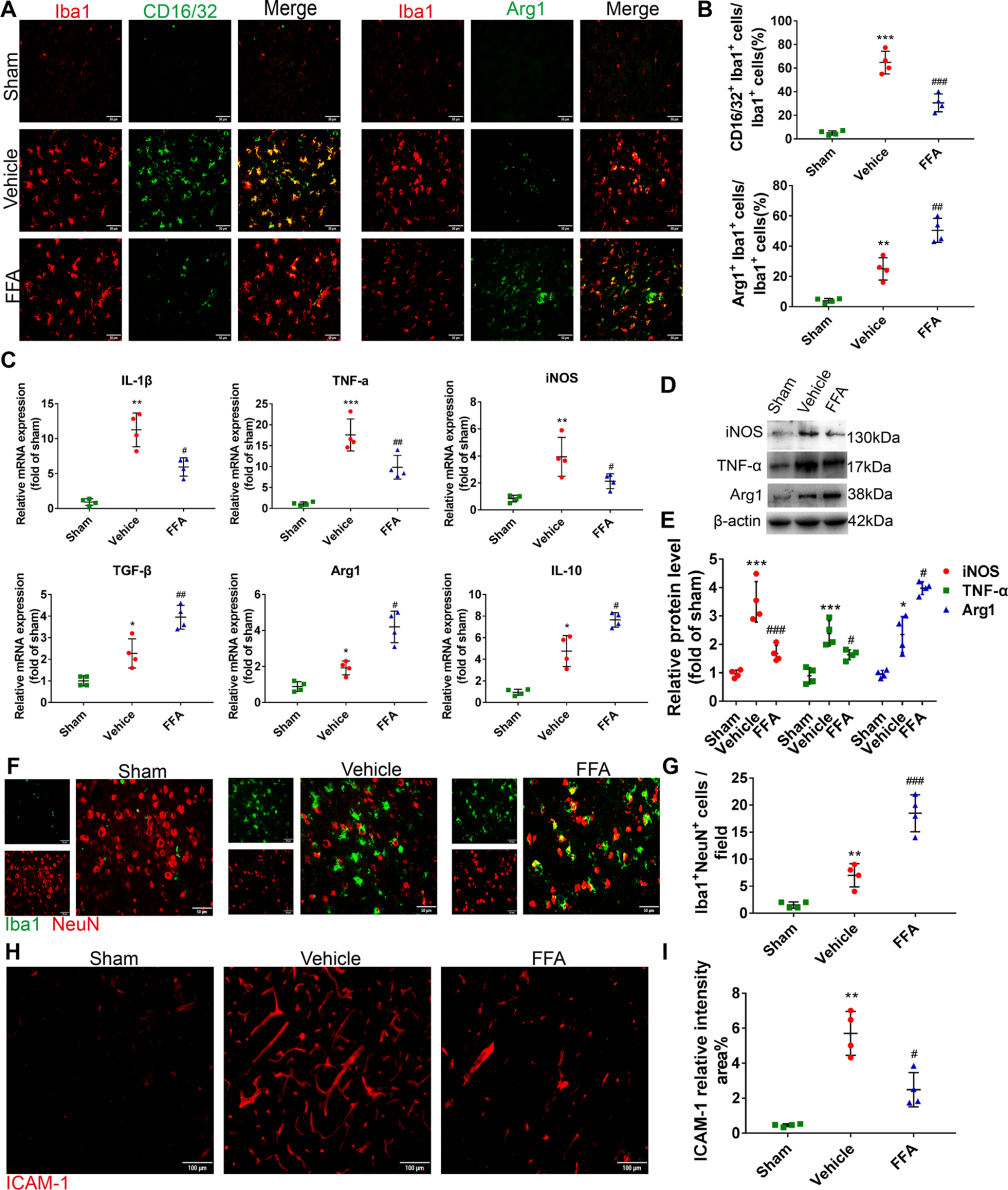
Effects of FFA on microglia/macrophage polarization after CA/CPR. **A** Representative photomicrographs of immunofluorescence staining for CD16/32 (green), Arg1 (green) and Iba1 (red) in the brains of the sham group and the experimental groups at 3 days after ROSC. Scale bar, 50 μm. **B** Quantification of the percentage of CD16/32 and Iba1 double-positive cells as well as Arg1 and Iba1 double-positive cells. **C** qPCR results of pro-inflammatory and anti-inflammatory markers in the brains of the sham group and the experimental groups at 3 days after ROSC. Western blot analysis of pro-inflammatory and anti-inflammatory markers in the brains of the sham group and the experimental groups at 3 days after ROSC (**D**) and the associated quantitative graph (**E**). **F** Representative photomicrographs of immunofluorescence staining for Iba1 (green) and NeuN (red) in the brains of the sham group and the experimental groups at 3 days after ROSC. Scale bar, 50 μm. **G** Quantification of the number of Iba1 and NeuN double-positive cells. **H** Representative photomicrographs of immunofluorescence staining for ICAM-1 in the brain of the sham group and the experimental groups at 3 days after ROSC. Scale bar, 100 μm. **I** Semi-quantitative results of ICAM-1 staining. *P < 0.05, **P < 0.01, ***P < 0.001 versus the sham group; #*P* < 0.05, ##*P* < 0.01, ### *P* < 0.001 versus the vehicle group, *n* = 4

In addition to BBB breakdown and the formation of brain edema, drastic neuroinflammation occurs following CA/CPR, leading to neurological deterioration [33]. Pro-inflammatory microglia/macrophages have been reported to engage in neuroinflammatory reactions and the subsequent destruction of the BBB [11]. We thus examined the polarization of microglia/macrophages, by colabeling the pro-inflammatory marker CD16/32 and the anti-inflammatory marker Arg1 with Iba1 and we measured the expression levels of molecules relevant to the pro-inflammatory response and inflammation resolution. In our preliminary study, we measured neuroinflammatory reactions, including pro-inflammatory microglia polarization and expression levels of inflammatory cytokines, on the first 3 days and day 7 after CA/CPR, and found that these changes seemed to peak on day 3 (Additional file 1: Fig. S2), we thus chose this time point for the detailed analysis. As shown, after CA/CPR, the percentage of CD16/32 and Iba1 double-positive microglia/macrophages was dramatically elevated in the vehicle group and was decreased in the FFA group (Fig. 6A, B). In contrast, the percentage of Arg1 and Iba1 double-positive microglia/macrophages was substantially increased in the FFA group (Fig. 6A, B). Similarly, FFA treatment significantly inhibited the mRNA levels of the pro-inflammatory markers *IL-1β*, *TNF-α*, and *iNOS* while enhancing the levels of the anti-inflammatory markers *TGF-β*, *Arg1*, and *IL-10* (Fig. 6C). Consistently, further Western blot analysis showed increased expression of Arg1 and decreased expression of TNF-α and iNOS after CA/CPR in brains treated with FFA compared with brains treated with vehicle (Fig. 6D, E).

Anti-inflammatory microglia/macrophages are associated with pro-phagocytic function toward damaged cells, which is essential for resolving inflammation. The clearance of damaged neurons was assessed by detecting the neuronal marker NeuN within Iba1^+^ microglia/macrophages. We found that FFA strengthened the phagocytic capacity of microglia/macrophages, manifesting as an increased number of Iba1^+^NeuN^+^ cells in the FFA group compared to the vehicle group (Fig. 6F, G). Additionally, FFA treatment was associated with significantly lower levels of ICAM-1 in the brain than the vehicle treatment (Fig. 6H, I). Hence, the neuroprotective effect of FFA was correlated with a reduced pro-inflammatory response and enhanced anti-inflammatory properties of microglia/macrophages.

#### FFA inhibited the upregulation of TRPM4 induced by CA/CPR in the neurovascular unit

**Fig. 7:**
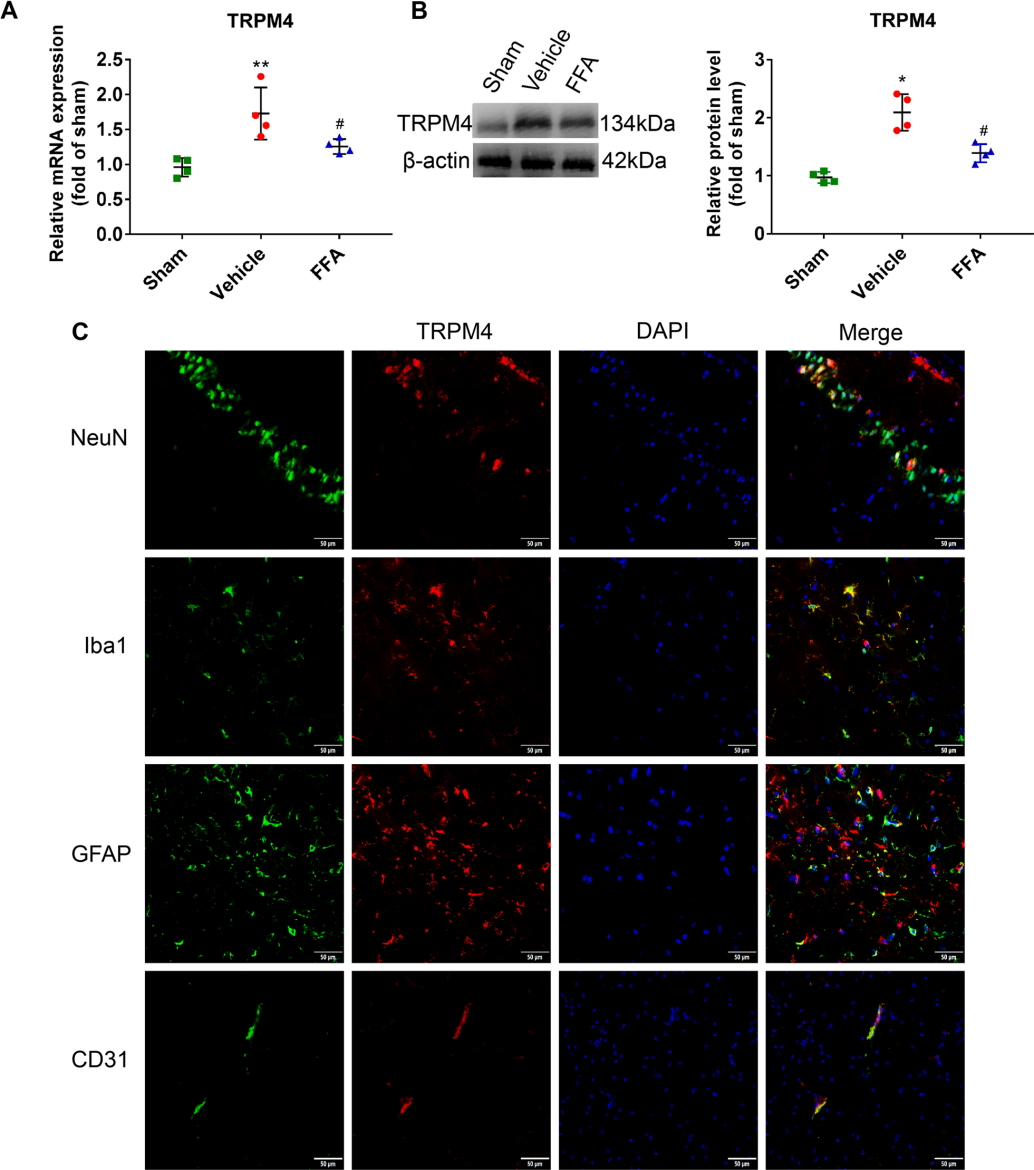
Effects of FFA on the TRPM4 channel in the neurovascular unit after CA/CPR. **A** qPCR results of *Trpm4* in the brains of the sham, vehicle and FFA groups at 24 h after ROSC. **B** Western blot analysis of TRPM4 in the brains of the sham group, vehicle and FFA groups at 24 h after ROSC and the associated quantitative graph. **C** Representative photomicrographs of immunofluorescence staining for TRPM4 (green) and several cell markers (red) including NeuN, Iba1, GFAP and CD31 in the brain at 24 h after ROSC. Scale bar, 50 μm. *P < 0.05, **P < 0.01 versus the sham group; #*P* < 0.05 versus the vehicle group, *n* = 4

Our previous studies have shown that glibenclamide, a selective inhibitor of SUR1, improved neurologic function after CA/CPR [18–20]. FFA has an established function in inhibiting the TRPM4 channel in the micromolar range [34]. We hypothesized that FFA may prime the brain to better withstand ischemic injury by blocking the overactivated TRPM4 channel. As expected, both the mRNA and protein levels of TRPM4 were increased at 24 h after ROSC (Fig. 7A, B). Double-immunofluorescence staining indicated that TRPM4 was expressed and localized in neurons (NeuN-positive), microglia (Iba1-positive), astrocytes (GFAP-positive) and endothelial cells (CD31-positive) (Fig. 7C). FFA treatment significantly inhibited the expression of TRPM4 after CA/CPR (Fig. 7A, B).

**Fig. 8:**
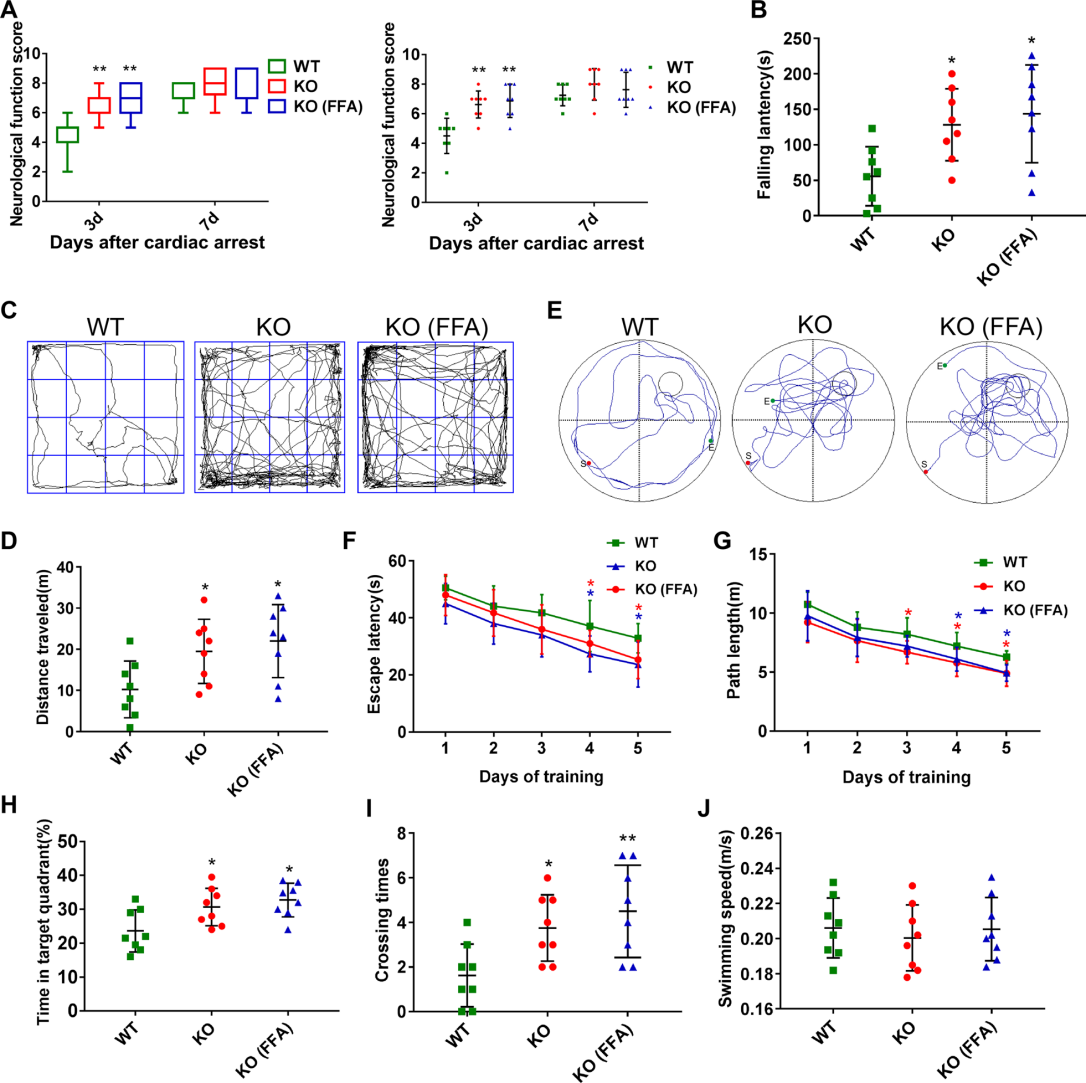
Effects of FFA on neurological function in *Trpm4*^*−/−*^ mice after CA/CPR. **A** Neurological function scores of survived mice from the WT, KO and KO (FFA) groups at 3 days and 7 days after ROSC. **B** The latency to drop from the accelerating rotarod. **C** Representative paths of mice during the open field test. **D** The total distance traveled in the open field test. **E** Representative swimming paths of mice during the probe trial. The latency to find the platform (**F**) and swimming distance (**G**) during the spatial exploration experiment. The percentage of time spent in the target quadrant (**H**) and platform crossovers (**I**) during the probe trial. **J** The swimming speed of mice in the Morris water maze test. *P < 0.05, **P < 0.01, ***P < 0.001 versus the WT group, *n* = 8. KO: vehicle-treated *Trpm4*^*−/−*^ mice; KO (FFA): flufenamic acid-treated *Trpm4*^*−/−*^ mice

**Fig. 9:**
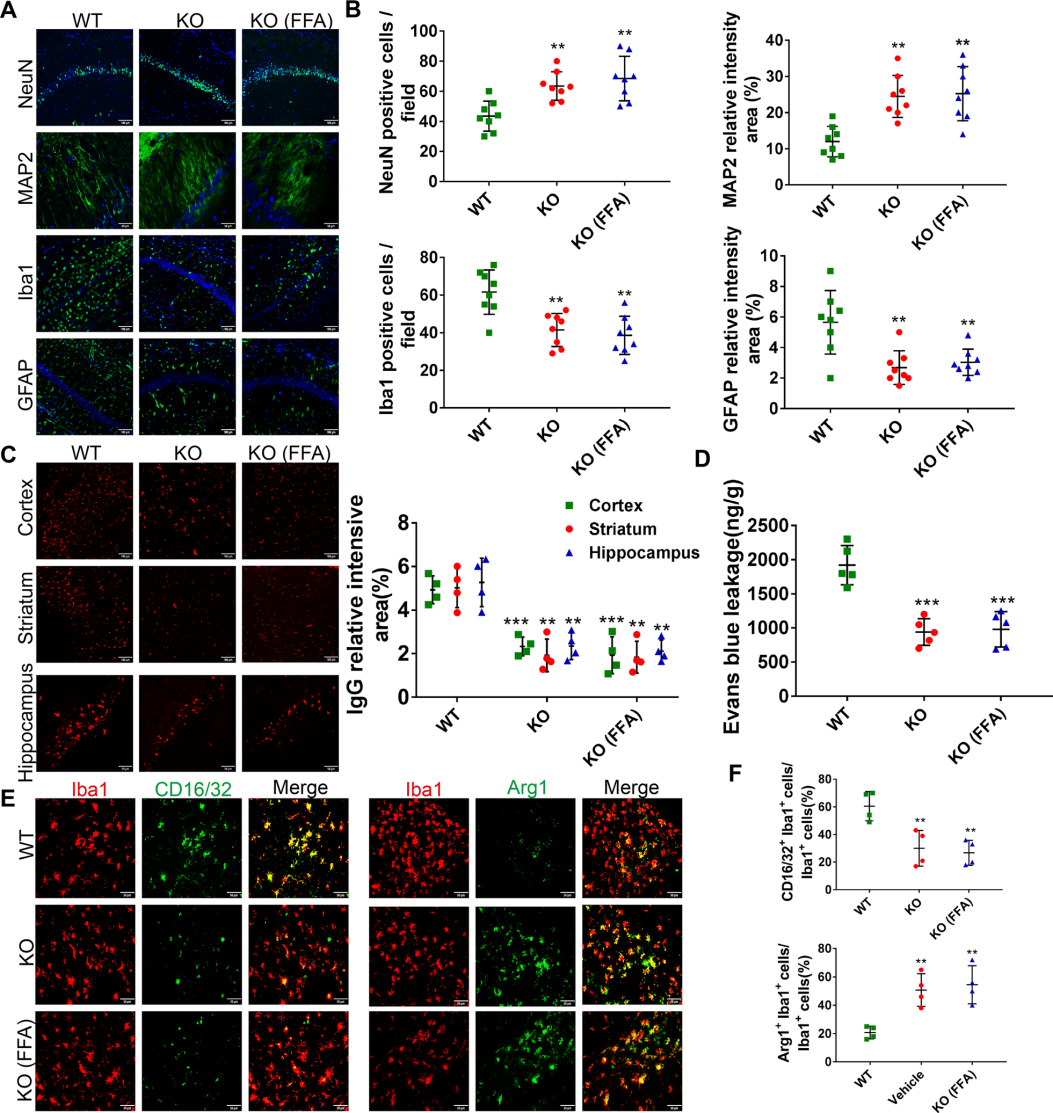
Effects of FFA on neuropathological damage, BBB integrity and microglia/macrophage polarization in *Trpm4*^*−/−*^ mice after CA/CPR. **A **Representative photomicrographs of post-cardiac arrest neuropathological damage characterized by immunofluorescence staining for NeuN, MAP2, GFAP, and Iba1 in the hippocampal CA1 region from each group at 7 days after ROSC. Scale bar, 100 μm or 50 μm. **B** Semi-quantitative results of NeuN, MAP2, Iba1, and GFAP staining. **C** Representative photomicrographs of immunofluorescence staining with IgG antibody (red) from each group at 3 days after ROSC and semi-quantitative results of IgG leakage. Scale bar, 100 μm or 50 μm. **D** Quantification of Evans blue leakage. **E** Representative photomicrographs of immunofluorescence staining for CD16/32 (green), Arg1 (green) and Iba1 (red) in the brains of each group at 3 days after ROSC. Scale bar, 50 μm. **F** Quantification of the percentage of CD16/32 and Iba1 double-positive cells as well as Arg1 and Iba1 double-positive cells. **P < 0.01, ***P < 0.001 versus the WT group, *n* = 4–8. KO: vehicle-treated *Trpm4*^*−/−*^ mice; KO (FFA): flufenamic acid-treated *Trpm4*^*−/−*^ mice

**Fig. 10:**
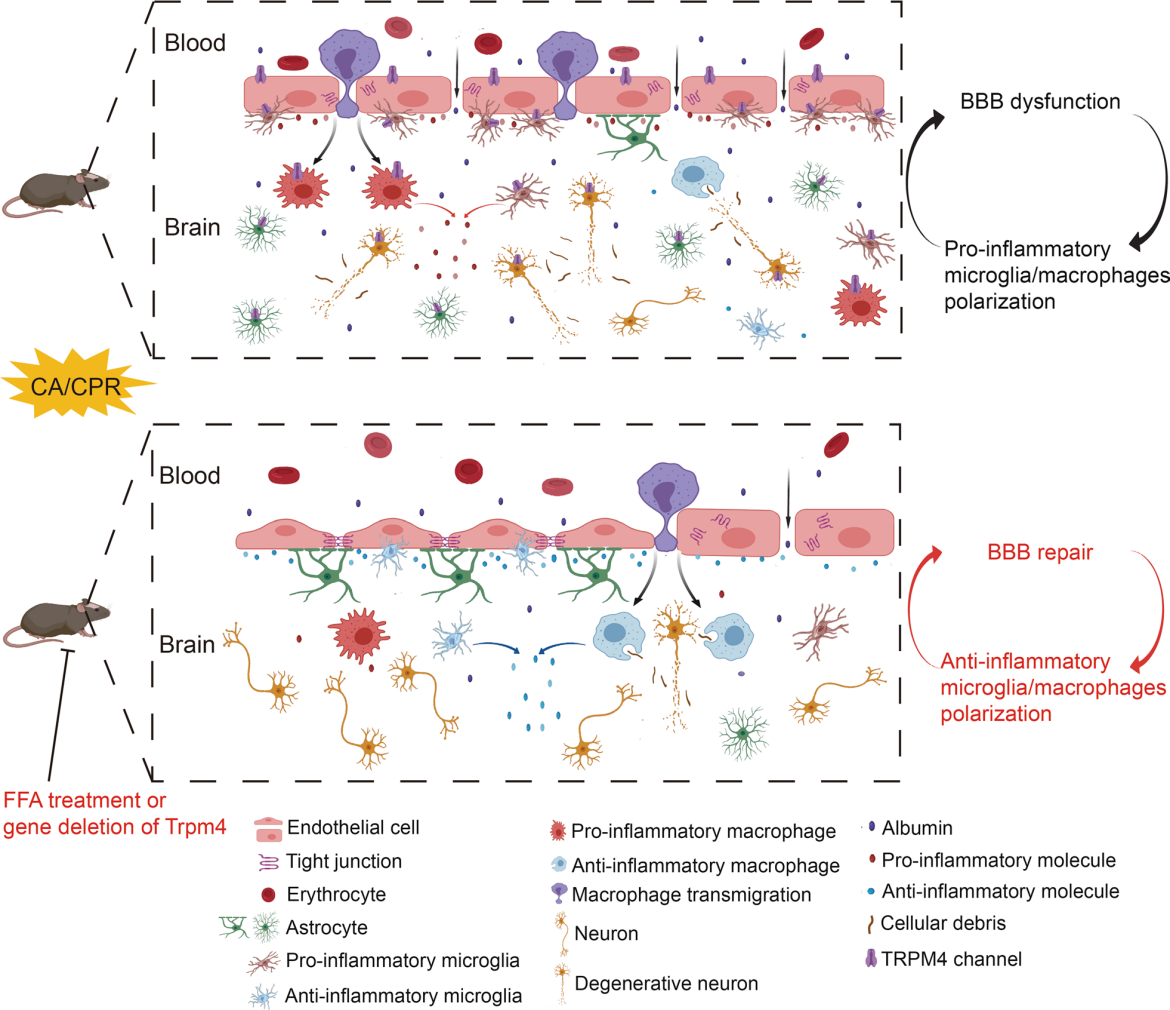
Schematic representation of the effects of FFA on post-CA brain injury. After cardiac arrest and cardiopulmonary resuscitation, the BBB is compromised due to ischemia/reperfusion, and microglia/macrophages switch toward the pro-inflammatory functional status. Both BBB breakdown and pro-inflammatory microglia/macrophages polarization form a vicious cycle contributing to enlarge brain injury continuously. FFA treatment effectively alleviates BBB breakdown, modifies the functional status of microglia/macrophages to enhance the removal of cellular debris and accelerate neuroinflammation resolution, and further mitigates neuronal injury, thus ultimately improving survival and neurologic outcome. Neuroprotection occurs at least in part through the modulation of the TRPM4 channel in the neurovascular unit. To sum up, FFA may stand out as a multipotent candidate drug for addressing brain injury resulting from cardiac arrest and cardiopulmonary resuscitation

To further verify the role of TRPM4 in the development of post-CA brain injury, we performed CA/CPR operations on *Trpm4*^*−/−*^ mice and observed the neurologic outcome and neuropathological manifestations. Compared with WT mice, *Trpm4*^*−/−*^ mice presented statistically higher neurological function scores, longer falling latencies in the rotarod test and longer traveling distances in the open field test after CA/CPR (Fig. 8A to D). Moreover, during the training period of the Morris water maze test, gene deletion of *Trpm4* substantially shortened the latency and swimming path to locate the hidden platform (Fig. 9F, G). In the probe phase, the blockage of TRPM4 increased the frequency of crossing the platform location and the time spent in the target quadrant (Fig. 9E, H, I). Nevertheless, no difference was observed in swimming speed among the three groups (Fig. 9J). Furthermore, in comparison to WT mice, *Trpm4*^*−/−*^ mice exhibited less histological damage, IgG leakage and Evans blue dye extravasation (Fig. 9A–D). Besides, *Trpm4*^*−/−*^ mice exhibited lower percentages of CD16/32^+^Iba1^+^ microglia/macrophages and higher percentages of Arg1^+^Iba1^+^ microglia/macrophages than WT mice (Fig. 9E, F). However, no additional neuroprotection was observed in *Trpm4*^*−/−*^ mice treated with FFA (Figs. 8, 9). These results, when taken together, proved that the TRPM4 channel was critically involved in brain injury after CA/CPR. Further, FFA exerted neuroprotection, at least in part by targeting the TRPM4 channel (Fig. 10).

## Discussion

Hypoxic ischemic brain injury after CA is the primary cause of death of CA survivors [35], thus it is imperative to develop an effective strategy to combat brain injury for CA victims with successful resuscitation. In this study, we investigated the neuroprotective effects of FFA and the underlying mechanisms after experimental CA/CPR in mice. We presented that FFA treatment remarkably improved 7-day survival and neurologic outcome, lessened neuropathological impairment, reduced brain edema and mitigated BBB breakdown. Additionally, pro-inflammatory microglia/macrophage polarization was suppressed, while anti-inflammatory microglia/macrophage polarization was promoted in CA/CPR mice administered with FFA. Lastly, we demonstrated that *Trpm4*^*−/−*^ mice exhibited comparable effects to FFA-treated mice, and *Trpm4*^*−/−*^ mice treated with FFA showed no benefit of superposition after CA/CPR. Taken together, our results supported that the neuroprotective effects of FFA against brain injury resulting from CA/CPR, at least partially depended on the inhibition of the overactivated TRPM4 channel in the neurovascular unit (Fig. 10). Our study highlighted the significance of TRPM4 in the development of post-CA brain injury and comprehensively evaluated the functions of FFA in post-CA brain injury.

Numerous studies have revealed that FFA can be an ion channel modulator [22, 23, 36, 37]. Since then, interest in the “off targets” of FFA (i.e., beyond its well-known effect of COX inhibition) protection has been rekindled, and the pleiotropic effects of FFA have been reevaluated. These intriguing findings gave rise to several studies indicating that FFA afforded neuroprotection in central nervous system (CNS) diseases in terms of dampening the inflammatory response, facilitating angiogenesis, preserving myelin and motor neurons, inhibiting glial activation and so on [24, 25]. Here, we stepped forward to elucidate the role of FFA in the model of CA/CPR. Our exciting findings in this study were that FFA treatment showed benefits in improving survival and neurological function and lessening neuropathological injuries following CA/CPR, which further supported the use of FFA as a lifesaving neuroprotectant to address CA/CPR-induced brain injury.

BBB disintegration is a threatening event in hypoxic ischemic encephalopathy after CA, causing fatal brain edema closely associated with poor prognosis. Osmotherapy is the conventional treatment for cerebral edema, by employing hypertonic saline or mannitol. However, under BBB dysfunction conditions, hypertonic saline or mannitol may accumulate within the brain, therefore potentiating edema formation. In fact, previous studies have indicated that hypertonic treatment fails to improve the outcome when administered after CA/CPR [38, 39]. Besides, osmotherapy is unlikely to alleviate, but may aggravate, the pro-inflammatory response of extravasated toxic substances [40]. From a clinical point of view, osmotherapy after CA/CPR remains an understudied topic due to these counterproductive effects. In this respect, preventing edema by enhancing BBB integrity is preferable to osmotherapy for treating the already swollen brain. Accumulating studies have demonstrated that targeting TRPM4 could be a new perspective for maintaining BBB integrity [14, 16, 41]. FFA was reported to ameliorate capillary fragmentation and secondary hemorrhage following spinal cord injury by blocking TRPM4 [42]. In the current study, we further provide encouraging evidence that FFA ameliorates BBB disruption and consequent edema formation following CA/CPR, supporting FFA as a promising agent in the rescue of BBB breakdown, which has important clinical implications given the considerable incidence rate of cerebral edema in CA/CPR patients.

Neuroinflammation plays a cardinal role in hypoxic ischemic encephalopathy resulting from CA/CPR [33]. Microglia may become activated for many weeks and develop a macrophage-like capacity to initiate inflammatory cascades of the CNS after CA/CPR. Classically activated microglia/macrophages secrete adverse cytokines that worsen brain injury and impede damaged tissue remodeling, whereas alternatively activated microglia/macrophages release anti-inflammatory mediators that hasten brain repair and potentiate the phagocytosis of dying cells [43]. Endowing activated microglia with a neuroprotective phenotype was found to improve outcomes in a model of CA/CPR [44, 45]. Moreover, a previous study demonstrated that microglia depletion by intrahippocampal injection of liposome-encapsulated clodronate was not sufficient to salvage neuronal degeneration after CA/CPR [46], which further supports that the balance between the numbers of reparative versus deleterious microglia/macrophages phenotypes rather than indiscriminate suppression of microglia activation may be instrumental in optimal brain repair and neurologic recovery. A recent study found that the administration of FFA inhibited microglia activation [24]. Herein, we add to the current knowledge that FFA transforms microglia/macrophages from a pro-inflammatory functional status into an anti-inflammatory status after CA/CPR. Strikingly, CA/CPR not only triggered an elevation in the percentage of pro-inflammatory microglia/macrophages, but also increased the percentage of anti-inflammatory microglia/macrophages, suggesting self-protection of the brain in response to CA/CPR. Previous studies also found that anti-inflammatory cytokines, such as TGF-β and IL-10 increased in the brain following CA/CPR [44, 47].

Sustained confrontation of dying cells is a major stimulus for harmful inflammatory reactions [48]. After CA/CPR, the selectively vulnerable regions with extensive cellular debris and cell corpses act as reservoirs for various cytotoxicities and pro-inflammatory cytokines, conducing to overwhelming neuroinflammation and the enlargement of secondary injury. In addition to triggering inflammation, necrotic tissue hampers neural reorganization and repair. Considering that interfering with apoptosis and necrosis is still difficult to achieve in the clinical real-world setting, the promotion of the reparative microglia/macrophages phenotype associated with augmented clearance function toward damaged cells and cellular debris may be indispensable for timely eliminating the source of inflammation and ultimately enabling effective functional recovery. We illustrated that the phagocytosis of microglia/macrophages was strengthened after FFA administration, which could be a reasonable explanation for the reduced accumulation of damaged cells in the brain. Besides, the adhesion molecule ICAM-1 is largely exposed due to glycocalyx degradation during CA/CPR, which promotes neuroinflammation by mediating leukocyte adhesion to the BBB and eventually transmigration to brain tissue [49]. We found that FFA treatment lowered the expression of ICAM-1, which contributed to the arrested amplification of neuroinflammation after CA/CPR. Altogether, our data reveal that FFA treatment after CA/CPR significantly optimizes the tissue-reparative function of microglia/macrophages to allow for better brain cleanup and a stronger capacity for neuroinflammation resolution, thereby averting further neuronal injury and favoring neurologic recovery.

The TRPM4 channel is a nonselective monovalent cation that is upregulated in a variety of CNS diseases [14, 17, 42] and leads to excessive influx of extracellular sodium, causing oncotic cell death and BBB breakdown, while gene deletion of this channel has been shown to afford neuroprotection in several models of neurological disease [13, 14]. Beyond its predominant effect of attenuating edema and preserving BBB function, increasing attention has been given to the study of the TRPM4 channel as a novel target for abating neuroinflammatory burden in several CNS diseases [50, 51]. Kurland et al. [21] indicated that in microglia in vivo and in vitro, gene silencing of *Trpm4* decreased pro-inflammatory gene expression following TLR4 activation in microglia, hinting at a delicate connection between TRPM4 and microglia polarization. In particular, the activation of TRPM4 requires calcium overload, which virtually occurs under conditions of CA/CPR [4]. In line with previous findings in the model of CA/CPR [19, 52], the expression of TRPM4 was upregulated and colocalized with the neurovascular unit. In the present study, we found more favorable neurologic outcome, milder histological injury, less IgG leakage, and more anti-inflammatory microglia/macrophage polarization in *Trpm4*^*−/−*^ mice after CA/CRR, which were comparable to the effects of FFA. Since FFA shows high potency in blocking the TRPM4 channel [22, 23], these results together further confirm the vital role of TRPM4 in the pathogenesis of post-CA brain injury. TRPM4, which can lead to catastrophic BBB disruption and a persistent neuroinflammatory response, could represent a promising and clinically relevant target for the future treatment of post-CA induced brain injury. As a step forward, we observed that FFA treatment significantly inhibited the upregulation of TRPM4, and FFA treatment combined with *Trpm4* deficiency showed no additive protective effect, which indicated that the suppression of TRPM4 in the neurovascular unit by FFA may be the main reason for better outcome following CA/CPR.

Although there is no doubt about the fundamental role of the endothelium in forming a barrier to restrict BBB permeability, the interaction of the endothelium with the components of the neurovascular unit should not be overlooked. In contrast to pericytes and astrocytes, the role of microglia in the regulation of BBB function is only beginning to be defined. Following ischemia, microglia form perivascular clusters, which display the intricate interplay between microglia and brain vessels. The communication of aggregated microglia with the endothelium exerts both beneficial and detrimental effects on BBB function depending on the microenvironment [53]. After BBB disruption, peripheral immune cells and toxic substances are recruited into the brain and whereby amplify neuroinflammation, including classically activated microglia/macrophage polarization [54]. These classically activated pro-inflammatory microglia may hinder BBB remodeling and result in vascular leakage, while anti-inflammatory microglia assist in the recovery of BBB damage [11]. Besides, neuroinflammation accompanying CNS diseases has been shown to accelerate BBB breakdown [55]. On this basis, the cross-talk between BBB dysfunction and microglia/macrophage polarization plays a key role in the vicious circle contributing to the pathogenesis of post-CA induced brain injury, which necessitates a conceptual shift from the widely recognized emphasis on protecting neurons to a novel treatment paradigm of targeting the entire neurovascular unit, especially microglia/macrophages. We propose that the effect of FFA on maintaining BBB integrity may not only be through directly suppressing TRPM4 on the endothelium, but also be derived from regulating the functional status of microglia/macrophages, namely that FFA treatment is an effective strategy to break the vicious cycle. Additionally, the pathophysiology of post-CA induced brain injury encompasses a heterogeneous cascade. Thus, using agents that affect multifaceted targets simultaneously may be more efficacious than modulating a single point to alleviate secondary brain injury after CA/CPR. We are convinced that FFA is promised to stand out as an attractive candidate drug for a polyvalent approach to stabilize the BBB following CA/CPR.

Apart from blocking the TRPM4 channel, FFA also affects the activity of other nonselective cationic channels [37]. In fact, specific pharmacological inhibition of TRPM4 is not practicable at present, probably due to structural similarities of TRPM4 to other channels. To date, pharmacological inhibition of TRPM4 by FFA has been pursued in several models [22–24]. Besides, TRPM4 has a relatively higher affinity for FFA than other ion channels [37]. It is suggested that low concentrations of FFA (~ 10 μM) may be suitable to determine the physiological effect of TRPM4 in situ, because it has little to no influence on other ion channels whose FFA sensitivity is much lower [37]. Accordingly, TRPM4 modulation may commonly account for the effects of FFA given its upregulation in the CA/CPR model and high sensitivity to FFA. Of note, plasma concentrations of 4–12 μM, measured in conditions of FFA clinical use, are sufficient to potently inhibit TRPM4 [56], which further support FFA as a TRPM4 inhibitor that could be translated into clinical use. To our knowledge, other channels potentially modulated by FFA are not expected to alleviate microvascular dysfunction, since this protective effect is specific for the TRPM4 channel. More importantly, as gene deletion of *Trpm4* mimicked the effect of FFA and no additional benefit was found in FFA-treated *Trpm4*^*−/−*^ mice, the participation of other ion channels is unlikely. Moreover, despite its primary characterization as a COX inhibitor, FFA showed lower effectiveness than other non-steroidal anti-inflammatory drugs, and it has been reported that FFA provides neuroprotection by inhibiting excitotoxicity and limiting neuroinflammation, which is independent of COX inhibition [25, 57]. Collectively, our findings corroborated that TRPM4 is the most likely regulator of the neuroprotective impact of FFA on CA/CPR-induced brain injury.

This study has some limitations. First, we did not study the effects of different doses of FFA on brain injury after CA/CPR. However, the FFA dose used in this study was derived from two previous well-designed studies that showed that FFA could inhibit TRPM4 and reduce spinal cord injury [24, 42]. Second, the lack of an additional protective effect of FFA in *Trpm4*^*−/−*^ mice may be due to the floor effect caused by FFA alone or Trpm4 knockout itself. Therefore, a direct observation of FFA inhibiting TRPM4 current in subsequent studies would provide more evidence. Nevertheless, we found that FFA did inhibit the upregulated expression of TRPM4, suggesting that the protective effect of FFA is at least partially mediated by inhibiting the TRPM4 channel.

## Conclusions

In summary, for the first time, we thoroughly characterized the therapeutic potential of FFA in post-CA brain injury, which includes the amelioration of BBB breakdown and the consequential formation of cerebral edema, the facilitation of a shift of microglia/macrophages toward a neuroprotective phenotype, and ultimately the mitigation of histological injury and improvement in functional outcome. These protective effects of FFA are possibly related to the inhibition of TRPM4. Our work provides novel insights into the mechanism of FFA-induced neuroprotection against brain injury caused by CA/CPR, advancing the current treatments and extending the clinical application of FFA beyond its typical use for analgesia. Although pharmacological inhibitors of TRPM4 have several issues, FFA appears to represent a multipotent and promising agent in the management of post-CA brain injury.

## Supplementary Information

**Additional file 1: Figure S1.** Results of agarose gel electrophoresis of tail DNA from WT and *Trpm4*^*−/−*^ mice. **Figure S2.** (A) Representative photomicrographs of immunofluorescence staining for CD16/32 (green) and Iba1 (red) in the brains in the sham and different CA/CPR groups (at day 1, day 2, day 3, and day 7 after ROSC, respectively). Scale bar, 50 μm. (B) Quantification of the percentage of CD16/32 and Iba1 double-positive cells. (C) qPCR results of inflammatory markers in the brains at at day 1, day 2, day 3, and day 7 after ROSC. *P < 0.05, **P < 0.01, ***P < 0.001 versus the sham group; #*P* < 0.05, ##*P* < 0.01, ###*P* < 0.001 versus the day 1 group; &*P* < 0.05, &&&*P* < 0.001 versus the day 2 group. n = 4. **Table S1.** Parameters of the vehicle and FFA groups at baseline and during post-CA care after ROSC. **Table S2.** Parameters of the WT, KO and KO (FFA) groups at baseline and during post-CA after ROSC.

## Data Availability

The datasets used and/or analyzed during this study are available from the corresponding authors on reasonable request.

## Abbreviations

CA: Cardiac arrest
CPR: Cardiopulmonary resuscitation
BBB: Blood–brain barrier
TRPM4: Transient receptor potential M4
SUR1-TRPM4: Sulfonylurea receptor 1-transient receptor potential M4
FFA: Flufenamic acid
*Trpm4*^*−/−*^: Gene deletion of *Trpm4*
WT: Wild-type
ECG: Electrocardiogram
ROSC: Return of spontaneous circulation
NeuN: Neuronal nuclei
Iba1: Ionized calcium-binding adapter molecule 1
GFAP: Glial fibrillary acidic protein
MAP2: Microtubule-associated protein 2
ICAM-1: Intercellular cell adhesion molecule-1
FJC: Fluoro-Jade C
TUNEL: Terminal deoxynucleotide transferase-mediated dUTP-biotin nick-end labeling
DWI: Diffusion-weighted imaging
IL: Interleukin
TNF-α: Tumor necrosis factor-α
iNOS: Inducible nitric oxide synthase
TGF-β: Transforming growth factor-β
Gapdh: Glyceraldehyde phosphate dehydrogenase
CNS: Central nervous system

## References

[1] Field JM, Hazinski MF, Sayre MR (2010). Part 1: Executive summary: 2010 American Heart Association Guidelines for Cardiopulmonary Resuscitation and Emergency Cardiovascular Care. Circulation.

[2] Shao F, Li CS, Liang LR (2014). Outcome of out-of-hospital cardiac arrests in Beijing. China Resuscitation.

[3] Myat A, Song KJ, Rea T (2018). Out-of-hospital cardiac arrest: current concepts. Lancet.

[4] Neumar RW, Nolan JP, Adrie C, et al. Post-cardiac arrest syndrome: Epidemiology, pathophysiology, treatment, and prognostication. A consensus statement from the International Liaison Committee on Resuscitation (American Heart Association, Australian and New Zealand Council on Resuscitation, European Resuscitation Council, Heart and Stroke Foundation of Canada, InterAmerican Heart Foundation, Resuscitation Council of Asia, and the Resuscitation Council of Southern Africa); The American Heart Association Emergency Cardiovascular Care Committee; The Council on Cardiovascular Surgery and Anesthesia; The Council on Cardiopulmonary, Perioperative, and Critical Care; The Council on Clinical Cardiology; And the Stroke Council. Circulation. 2008; 118: 2452–2483. 10.1161/CIRCULATIONAHA.108.190652 18948368

[5] Neumar RW (2000). Molecular mechanisms of ischemic neuronal injury. Ann Emerg Med.

[6] Sekhon MS, Ainslie PN, Griesdale DE (2017). Clinical pathophysiology of hypoxic ischemic brain injury after cardiac arrest: A “two-hit” model. Crit Care.

[7] Gueugniaud PY, Garcia-Darennes F, Gaussorgues P (1991). Prognostic significance of early intracranial and cerebral perfusion pressures in post-cardiac arrest anoxic coma. Intensive Care Med.

[8] Chae MK, Ko E, Lee JH (2016). Better prognostic value with combined optic nerve sheath diameter and grey-to-white matter ratio on initial brain computed tomography in post-cardiac arrest patients. Resuscitation.

[9] Naito H, Isotani E, Callaway CW (2016). Intracranial pressure increases during rewarming period after mild therapeutic hypothermia in post cardiac arrest patients. Ther Hypothermia Temp Manag.

[10] Wagner KR, Dean C, Beiler S (2005). Plasma infusions into porcine cerebral white matter induce early edema, oxidative stress, pro-inflammatory cytokine gene expression and DNA fragmentation: Implications for white matter injury with increased blood-brain-barrier permeability. Curr Neurovasc Res.

[11] Ronaldson PT, Davis TP (2020). Regulation of blood–brain barrier integrity by microglia in health and disease: A therapeutic opportunity. J Cereb Blood Flow Metab.

[12] Vennekens R, Nilius B (2007). Insights into TRPM4 function, regulation and physiological role. Handb Exp Pharmacol.

[13] Chen X, Liu K, Lin Z (2020). Knockout of transient receptor potential melastatin 4 channel mitigates cerebral edema and neuronal injury after status epilepticus in mice. J Neuropathol Exp Neurol.

[14] Wang X, Chang Y, He Y (2020). Glimepiride and glibenclamide have comparable efficacy in treating acute ischemic stroke in mice. Neuropharmacology.

[15] Lee JY, Choi HY, Na WH (2014). Ghrelin inhibits BSCB disruption/hemorrhage by attenuating MMP-9 and SUR1/TrpM4 expression and activation after spinal cord injury. Biochim Biophys Acta Mol Basis Dis.

[16] Jiang B, Li L, Chen Q (2017). Role of glibenclamide in brain injury after intracerebral hemorrhage. Transl Stroke Res.

[17] Tosun C, Kurland DB, Mehta R (2013). Inhibition of the Sur1-Trpm4 channel reduces neuroinflammation and cognitive impairment in subarachnoid hemorrhage. Stroke.

[18] Huang K, Gu Y, Hu Y (2015). Glibenclamide improves survival and neurologic outcome after cardiac arrest in rats. Crit Care Med.

[19] Huang K, Wang Z, Gu Y (2016). Glibenclamide is comparable to target temperature management in improving survival and neurological outcome after asphyxial cardiac arrest in rats. J Am Heart Assoc.

[20] Huang K, Wang Z, Gu Y (2018). Glibenclamide prevents water diffusion abnormality in the brain after cardiac arrest in rats. Neurocrit Care.

[21] Kurland DB, Gerzanich V, Karimy JK (2016). The Sur1-Trpm4 channel regulates NOS2 transcription in TLR4-activated microglia. J Neuroinflamm.

[22] Simard C, Sallé L, Rouet R (2012). Transient receptor potential melastatin 4 inhibitor 9-phenanthrol abolishes arrhythmias induced by hypoxia and re-oxygenation in mouse ventricle. Brit J Pharmacol.

[23] Guinamard R, Demion M, Magaud C (2006). Functional expression of the TRPM4 cationic current in ventricular cardiomyocytes from spontaneously hypertensive rats. Hypertension.

[24] Yao Y, Xu J, Yu T (2018). Flufenamic acid inhibits secondary hemorrhage and BSCB disruption after spinal cord injury. Theranostics.

[25] Daniels MJD, Rivers-Auty J, Schilling T (2016). Fenamate NSAIDs inhibit the NLRP3 inflammasome and protect against Alzheimer’s disease in rodent models. Nat Commun.

[26] Fernández M, Lao-Peregrín C, Martín ED (2010). Flufenamic acid suppresses epileptiform activity in hippocampus by reducing excitatory synaptic transmission and neuronal excitability. Epilepsia.

[27] Hayashida K, Bagchi A, Miyazaki Y (2019). Improvement in outcomes after cardiac arrest and resuscitation by inhibition of S-Nitrosoglutathione reductase. Circulation.

[28] Kida K, Minamishima S, Wang H (2012). Sodium sulfide prevents water diffusion abnormality in the brain and improves long term outcome after cardiac arrest in mice. Resuscitation.

[29] Li M, Chen S, Shi X (2018). Cell permeable HMGB1-binding heptamer peptide ameliorates neurovascular complications associated with thrombolytic therapy in rats with transient ischemic stroke. J Neuroinflamm.

[30] Zhu J, Li Z, Ji Z (2022). Glycocalyx is critical for blood-brain barrier integrity by suppressing caveolin1-dependent endothelial transcytosis following ischemic stroke. Brain Pathol.

[31] Zhu J, Liu K, Huang K (2018). Metformin improves neurologic outcome via AMP-activated protein kinase-mediated autophagy activation in a rat model of cardiac arrest and resuscitation. J Am Heart Assoc.

[32] Hazelton JL, Balan I, Elmer GI (2010). Hyperoxic reperfusion after global cerebral ischemia promotes inflammation and long-term hippocampal neuronal death. J Neurotrauma.

[33] Liu F, McCullough LD (2013). Inflammatory responses in hypoxic ischemic encephalopathy. Acta Pharmacol Sin.

[34] Guinamard R, Sallé L, Simard C (2011). The non-selective monovalent cationic channels TRPM4 and TRPM5. Adv Exp Med Biol.

[35] Laver S, Farrow C, Turner D (2004). Mode of death after admission to an intensive care unit following cardiac arrest. Intensive Care Med.

[36] Ullrich ND, Voets T, Prenen J (2005). Comparison of functional properties of the Ca2+-activated cation channels TRPM4 and TRPM5 from mice. Cell Calcium.

[37] Guinamard R, Simard C, Del Negro C (2013). Flufenamic acid as an ion channel modulator. Pharmacol Therapeut.

[38] Noppens RR, Kelm RF, Lindemann R (2012). Effects of a single-dose hypertonic saline hydroxyethyl starch on cerebral blood flow, long-term outcome, neurogenesis, and neuronal survival after cardiac arrest and cardiopulmonary resuscitation in rats. Crit Care Med.

[39] Breil M, Krep H, Heister U (2012). Randomised study of hypertonic saline infusion during resuscitation from out-of-hospital cardiac arrest. Resuscitation.

[40] Burks SR, Kersch CN, Witko JA (2021). Blood-brain barrier opening by intracarotid artery hyperosmolar mannitol induces sterile inflammatory and innate immune responses. Proc Natl Acad Sci U S A.

[41] Lin Z, Huang H, Gu Y (2017). Glibenclamide ameliorates cerebral edema and improves outcomes in a rat model of status epilepticus. Neuropharmacology.

[42] Gerzanich V, Woo SK, Vennekens R (2009). De novo expression of Trpm4 initiates secondary hemorrhage in spinal cord injury. Nat Med.

[43] David S, Kroner A (2011). Repertoire of microglial and macrophage responses after spinal cord injury. Nat Rev Neurosci.

[44] Wang J, Fujiyoshi T, Kosaka Y (2013). Inhibition of soluble epoxide hydrolase after cardiac arrest/cardiopulmonary resuscitation induces a neuroprotective phenotype in activated microglia and improves neuronal survival. J Cereb Blood Flow Metab.

[45] Grace PM, Shimizu K, Strand KA (2015). (+)-Naltrexone is neuroprotective and promotes alternative activation in the mouse hippocampus after cardiac arrest/cardiopulmonary resuscitation. Brain Behav Immun.

[46] Drabek T, Janata A, Jackson EK (2012). Microglial depletion using intrahippocampal injection of liposome-encapsulated clodronate in prolonged hypothermic cardiac arrest in rats. Resuscitation.

[47] Jiang M, Li R, Lyu J (2020). MCC950, a selective NLPR3 inflammasome inhibitor, improves neurologic function and survival after cardiac arrest and resuscitation. J Neuroinflamm.

[48] Liesz A, Dalpke A, Mracsko E (2015). DAMP signaling is a key pathway inducing immune modulation after brain injury. J Neurosci.

[49] Zhu J, Li X, Yin J (2018). Glycocalyx degradation leads to blood–brain barrier dysfunction and brain edema after asphyxia cardiac arrest in rats. J Cereb Blood Flow Metab.

[50] Makar TK, Gerzanich V, Nimmagadda VKC (2015). Silencing of Abcc8 or inhibition of newly upregulated Sur1-Trpm4 reduce inflammation and disease progression in experimental autoimmune encephalomyelitis. J Neuroinflamm.

[51] Schattling B, Steinbach K, Thies E (2012). TRPM4 cation channel mediates axonal and neuronal degeneration in experimental autoimmune encephalomyelitis and multiple sclerosis. Nat Med.

[52] Nakayama S, Taguchi N, Isaka Y (2018). Glibenclamide and therapeutic hypothermia have comparable effect on attenuating global cerebral edema following experimental cardiac arrest. Neurocrit Care.

[53] Haruwaka K, Ikegami A, Tachibana Y (2019). Dual microglia effects on blood brain barrier permeability induced by systemic inflammation. Nat Commun.

[54] Bernstein DL, Zuluaga-Ramirez V, Gajghate S (2020). MiR-98 reduces endothelial dysfunction by protecting blood-brain barrier (BBB) and improves neurological outcomes in mouse ischemia/reperfusion stroke model. J Cereb Blood Flow Metab.

[55] Obermeier B, Daneman R, Ransohoff RM (2013). Development, maintenance and disruption of the blood-brain barrier. Nat Med.

[56] Aly FA, Al-Tamimi SA, Alwarthan AA (2000). Determination of flufenamic acid and mefenamic acid in pharmaceutical preparations and biological fluids using flow injection analysis with tris(2,2′-bipyridyl)ruthenium(II) chemiluminescence detection. Anal Chim Acta.

[57] Khansari PS, Halliwell RF (2019). Mechanisms underlying neuroprotection by the NSAID mefenamic acid in an experimental model of stroke. Front Neurosci.

