# Supplementary material for: Flufenamic acid improves survival and neurologic outcome after successful cardiopulmonary resuscitation in mice
Source: J Neuroinflammation. 2022 Sep 1;19:214. doi: 10.1186/s12974-022-02571-2 (PMC9438280; doi:10.1186/s12974-022-02571-2)
Supplement: Supplementary file 1 — Additional file 1: Figure S1. Results of agarose gel electrophoresis of tail DNA from WT and Trpm4−/− mice. Figure S2. (A) Representative photomicrographs of immunofluorescence staining for CD16/32 (green) and Iba1 (red) in the brains in the sham and different CA/CPR groups (at day 1, day 2, day 3, and day 7 after ROSC, respectively). Scale bar, 50 μm. (B) Quantification of the percentage of CD16/32 and Iba1 double-positive cells. (C) qPCR results of inflammatory markers in the brains at at day 1, day 2, day 3, and day 7 after ROSC. *P < 0.05, **P < 0.01, ***P < 0.001 versus the sham group; #P < 0.05, ##P < 0.01, ###P < 0.001 versus the day 1 group; &P < 0.05, &&&P < 0.001 versus the day 2 group. n = 4. Table S1. Parameters of the vehicle and FFA groups at baseline and during post-CA care after ROSC. Table S2. Parameters of the WT, KO and KO (FFA) groups at baseline and during post-CA after ROSC. [file 12974_2022_2571_MOESM1_ESM.pdf]

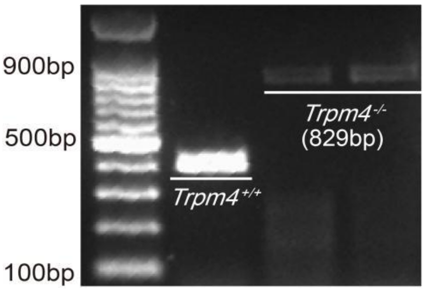

Supplemental Figure 1. Results of agarose gel electrophoresis of tail DNA from WT and *Trpm4$^{-/-}$* mice.

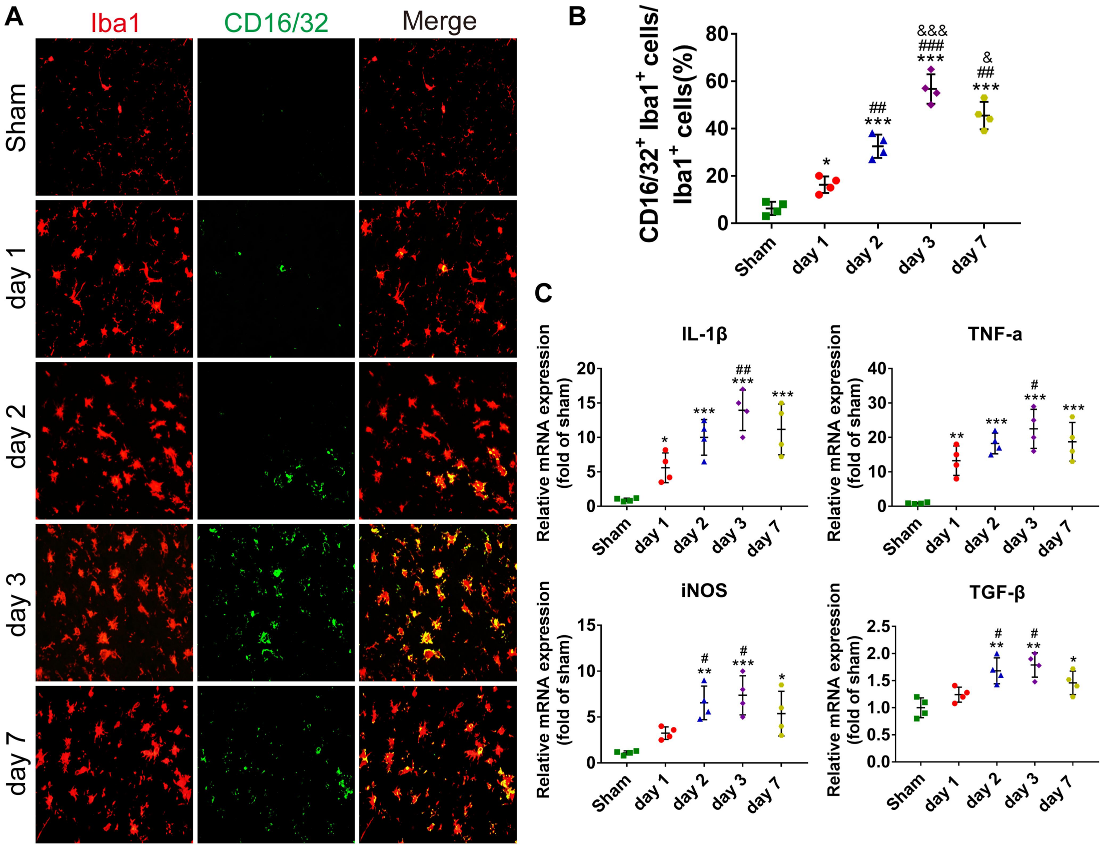

Supplemental Figure 2. Dynamic changes in pro-inflammatory microglia/macrophage polarization and mRNA expression of inflammatory markers following CA/CPR.

(**A**) Representative photomicrographs of immunofluorescence staining for CD16/32 (green) and Iba1 (red) in the brains in the sham and different CA/CPR groups (at day 1, day 2, day 3, and day 7 after ROSC, respectively). Scale bar, 50 μm. (**B**) Quantification of the percentage of CD16/32 and Iba1 double-positive cells. (**C**) qPCR results of inflammatory markers in the brains at at day 1, day 2, day 3, and day 7 after ROSC. **P*<0.05, ***P*<0.01, ****P*<0.001 versus the sham group; #*P*<0.05, ##*P*<0.01, ###*P*<0.001 versus the day 1 group; &*P*<0.05, &&&*P*<0.001 versus the day 2 group. n = 4.

Supplemental Table 1. Parameters of the vehicle and FFA groups at baseline and during post-CA care after ROSC.

Parameters	Time points	Vehicle	FFA
Body weight, g	Baselines	26.35 ± 1.08	26.74 ± 1.09
Time required for ROSC, s	Baselines	124.93 ± 17.17	130.05 ± 15.07
Total dose of epinephrine, μg	Baselines	8.34 ± 0.57	8.18 ± 0.46
Heart rate, beats/min	Baselines	536.63 ± 14.89	531.73 ± 12.14
	10 minutes	504.78 ± 12.83	509.78 ± 14.06
	30 minutes	551.30 ± 14.90	547.78 ± 10.98
	60 minutes	541.90 ± 12.46	537.55 ± 8.04

Supplemental Table 2. Parameters of the WT, KO and KO (FFA) groups at baseline and during post-CA after ROSC.

Parameters	Time points	WT	KO	KO (FFA)
Body weight, g	Baselines	26.29 ± 0.78	25.95 ± 0.61	26.18 ± 0.67
Time required for ROSC, s	Baselines	123.36 ± 12.48	128.86 ± 14.36	132.14 ± 15.51
Total dose of epinephrine, μg	Baselines	8.40 ± 0.61	8.27 ± 0.49	8.45 ± 0.60
Heart rate, beats/min	Baselines	530.36 ± 14.70	524.29 ± 18.69	522.00 ± 11.72
	10 minutes	510.07 ± 15.65	505.78 ± 19.15	512.14 ± 10.60
	30 minutes	541.14 ± 18.75	543.79 ± 14.69	550.57 ± 13.83
	60 minutes	540.36 ± 14.35	530.43 ± 18.32	533.07 ± 17.87
